# Acupuncture for neuropathic pain: A meta-analysis of randomized control trials

**DOI:** 10.3389/fneur.2022.1076993

**Published:** 2023-01-09

**Authors:** Zitong Feng, Shaoyang Cui, Huijun Yang, Yixiao Wang, Xuan Zhou, John Wong, Liting Lai, Zeyu Yang, Bingjing Huang, Huiyan Zheng, Mingzhu Xu

**Affiliations:** ^1^Medical College of Acu-Moxi and Rehabilitation, Guangzhou University of Chinese Medicine, Guangzhou, China; ^2^Department of Rehabilitation, Shenzhen Hospital of Guangzhou University of Chinese Medicine, Shenzhen, China; ^3^Shenzhen Hospital, Southern Medical University, Shenzhen, China; ^4^Formula-Pattern Research Center, School of Traditional Chinese Medicine, Jinan University, Guangzhou, China; ^5^School of Nursing, MGH Institute of Health Professions, Boston, MA, United States; ^6^Department of Occupational Therapy, MGH Institute of Health Professions, Boston, MA, United States

**Keywords:** acupuncture, neuropathic pain, alternative and complementary medicine, systematic review, meta-analysis

## Abstract

**Background:**

Neuropathic pain (NP) is expected to increase due to the high risk of global population aging. Acupuncture has a definite clinical effect on NP. Therefore, a systematic review and meta-analysis were conducted to evaluate the effect on pain intensity and safety of acupuncture in patients with NP.

**Methods:**

An encompassing search of specific authoritative databases in English, from their inception to 2022, was performed. The databases were as follows: Scopus, Ovid EMBASE, Ovid Cochrane Database of Systematic Reviews, Ovid Cochrane Central Register of Controlled Trials, Ovid MEDLINE(R) and Epub Ahead of Print, In-Process and Other Non-Indexed Citations, and Daily. All the randomized controlled trials regarding the acupuncture treatment of NP will be included. Methodological quality assessment of the included trials was assessed based on the risk of bias from the Cochrane handbook. A meta-analysis was performed for the main outcomes. In addition, sensitivity analysis, subgroup analysis, and funnel plot were also carried out.

**Results:**

A total of 16 studies with 1,021 patients with NP were evaluated in a systematic review. According to the results of the overall meta-analysis in eight RCTs with 338 participants, the acupuncture group was better than the control group in improving changes in pain intensity (SMD −0.59, 95% CI: −0.95 to −0.23, *P* = 0.001). In subgroup analysis, five trials indicated that acupuncture was more effective in improving changes in pain intensity than sham acupuncture (SMD −0.54, 95% CI: −0.95 to −0.13, *P* = 0.01), two trials evaluated the effect on changes in pain intensity in the comparison of acupuncture and conventional treatments, no significant difference existed (SMD −0.61, 95% CI: −1.83 to 0.61, *P* = 0.33), and one trial compared acupuncture with blank control evaluating the effect of changes in pain intensity with a significant difference. Eleven studies mentioned the safety conditions and acupuncture-induced AEs were mild and reversible. Both the sensitivity analysis and funnel plot analysis showed that the meta-analysis was stable and irreversible without publication bias. The GRADE was rated as “very low.”

**Conclusion:**

The acupuncture group had higher effectiveness than sham intervention or blank control for changes in pain intensity, but there is no significant difference between acupuncture and conventional treatments in treating NP. The acupuncture-induced adverse events were mild and reversible. However, the interpretation of our results should be performed cautiously due to the low methodological quality of selected publications.

**Systematic review registration:**

https://www.crd.york.ac.uk/prospero/display_record.php?ID=CRD42022306461.

## Introduction

The International Association for the Study of Pain (IASP) recently updated the definition of neuropathic pain (NP) as “pain caused by a lesion or disease of the somatosensory nervous system” (1). NP has both the “positive” symptoms (hyperalgesia, allodynia, shooting pain, burning pain, and especially at rest) that require therapy and the “negative” symptoms (sensory deficits such as hypalgesia and hypesthesia) that cannot be treated with medication (2). A survey of the general population sampled by multimodal recruitment in 2017 reported that about 10% of people in the United States suffered from NP (3). With the aging of the global population, NP is extremely likely to increase (4). Moreover, NP seriously affects patients' quality of life (5). Specifically, patients with NP have anxiety, depression, poor sleep, psychological disorder, physical disability, and social dysfunction (6–10). An observational study on the economic burden of patients with NP at all pain intensity levels in the United States showed that the annualized direct medical expenses to payers were $6,016, the annualized direct expenses to subjects were $2,219, and the annualized indirect expenses of each subject were $19,000 (11).

To date, the medications of NP focus on five categories including serotonin/norepinephrine-modulating antidepressants, Na-blocker anticonvulsants, Ca-modulator anticonvulsants, tramadol, and opioids, and two types of topical medicine including local anesthetics and capsaicin (12). However, pharmacological treatment is not very effective for NP for the reason that the patients keep reporting inadequate pain relief, and a progressive decrease in the estimated effect of NP drugs has been reported (13). In addition, a randomized controlled trial (RCT) on the safety of antiepileptics and antidepressants for NP showed that the incidence of any treatment-emergent adverse event (AE) ranged from 7 to 91.7% compared with the placebo groups, and the dizziness, drowsiness, nausea, and constipation were the most commonly reported AEs (14). Despite the lack of evidence to show beneficial effects, clinical trials on novel analgesic medicine to treat NP are lacking in recent years (15, 16).

Given the situation that NP is mostly chronic, which means that long-term management is required, therefore, it is critical to developing a treatment protocol concentrating on improving efficacy and safety monitoring is critical (17). As an alternative and complementary medicine, acupuncture refers to inserting needles into acupoints or specific parts of the human body at different depths by various manipulations (18). Widespread and effective applications of acupuncture were encouraged in the clinical treatment of NP in recent years (19–27). In addition, a meta-analysis reported that acupuncture could be considered the safer therapy in medications, with the reason that serious AEs were rare, and the most common AEs were mild (28).

However, some researchers consider the difference between acupuncture and sham acupuncture as not being clinically significant (29). A systematic review and meta-analysis (30) on acupuncture in the treatment of NP in adults published in 2017 suggested that it is challenging to either support or refute the effect of acupuncture for NP due to limited data available. Considering the widespread and effective applications of acupuncture in the clinical treatment of NP, the previous meta-analysis conclusions need to be further verified. Therefore, this study was conducted to explore the effect on pain intensity and safety of acupuncture in patients with NP.

## Materials and methods

The protocol has been registered in the PROSPERO database with registration number: CRD42022306461. This study was carried out based on the Preferred Reporting Items for Systematic Reviews and Meta-Analyses (PRISMA) guidelines updated in 2020 (31). A detailed description of the PRISMA 2020 checklist of this study is provided in the Supplementary material.

### Search strategy

An encompassing search of specific authoritative databases was conducted. The search strategy was designed and performed by a professional librarian. The procedure was described in the Supplementary material of this article. The databases were as follows:

1. EBM Reviews—Cochrane Central Register of Controlled Trials April 2022;
2. EBM Reviews—Cochrane Database of Systematic Reviews 2005 to 5 May 2022;
3. Embase 1974 to 4 May 2022;
4. Ovid MEDLINE(R) and Epub Ahead of Print, In-Process, In-Data-Review and Other Non-Indexed Citations, Daily and Versions 1946 to 4 May 2022.

### Selection criteria

Studies were eligible if met the following criteria: (1) RCTs evaluating acupuncture for NP; (2) participants with the diagnosis of NP; (3) acupuncture treatments as the main observational therapies [including traditional acupuncture, electroacupuncture (EA), auricular acupuncture, and abdominal acupuncture]; (4) the control group could be conventional treatment, sham acupuncture (close to the acupoints but not penetrating the skin), or blank control (no intervention during the treatment period); and (5) pain change variables including but not limited to visual analog scale (VAS) score, numeric rating scale (NRS), and Brief Pain Inventory-Short Form (BPI-SF) worst pain score. Limited to reports in English, AEs were used to assess the safety of acupuncture therapies. The following studies were excluded: (1) conference abstracts, case reports, protocols, reviews, and animal or cellular level experiments; (2) duplicated literature; (3) studies with insufficient data; (4) trials with acupuncture therapy in the control group; (5) studies using methods not based on Traditional Chinese Medicine (TCM) theory like a dry needle; (6) articles on moxibustion, cupping, herbal medicine, laser acupuncture, and any combination of the above; and (7) literature not published in English.

Full-text articles were retrieved after screening based on titles and abstracts of all articles, according to the criteria by two independent researchers. In addition, the discussion was carried out in case of disagreements, and a third party helped to reach a consensus if necessary.

### Data extraction

Data were collected independently by two researchers using Excel tables from every included study and reviewed by a third party. The collected information contained the first author's name, year of publication, subjects, age, condition, sample size, interventions, sessions, outcome measures, follow-up, and the selected acupoints of treatment. The pain intensity outcomes were recorded as continuous variables. For each study, the mean difference (MD) before and after treatment was used to pool differences between experimental and control groups.

### Risk of bias assessment

Studies were evaluated with the Cochrane risk of bias assessment tool (32) by the two researchers independently. Related evaluations were as follows: sequence generation, allocation concealment, blinding, incomplete outcome data, selective outcome reporting, and other sources. Moreover, each part of the evaluation was defined as low, high, or unclear risk of bias. A third party helps reach a consensus if there was a conflict.

### Statistical analysis

All analyses were carried out by RevMan 5.3. Qualitative analysis was carried out if extraction was insufficient to conduct a meta-analysis. Given the strong relevance among the scales of pain assessment (33), the results of the NRS, VAS, and BPI-SF worst pain score were used in the meta-analysis. Outcome data were performed with the standardized mean difference (SMD) and 95% confidence interval (CI) to standardize the study results into a unified scale. SMD with 95% CI was calculated with heterogeneity tested by the *I*^2^ test. Data was combined by a fixed effect model when *I*^2^ < 50%. Otherwise, a random effect model was carried out. There was a significant difference if the *p*-value was < 0.05 between the two groups. Subgroup analysis or sensitivity analysis could help find out the sources of heterogeneity. Furthermore, a descriptive analysis was conducted when the reasons for heterogeneity could not be determined. A funnel plot was applied to assess publication bias.

### Quality of evidence

We rated the general quality of outcome with the classification of GRADEpro GDT (https://www.gradepro.org/) in the following areas: study design, risk of bias, inconsistency, indirectness, imprecision, and other considerations.

## Results

### Study selection

**Figure 1:**
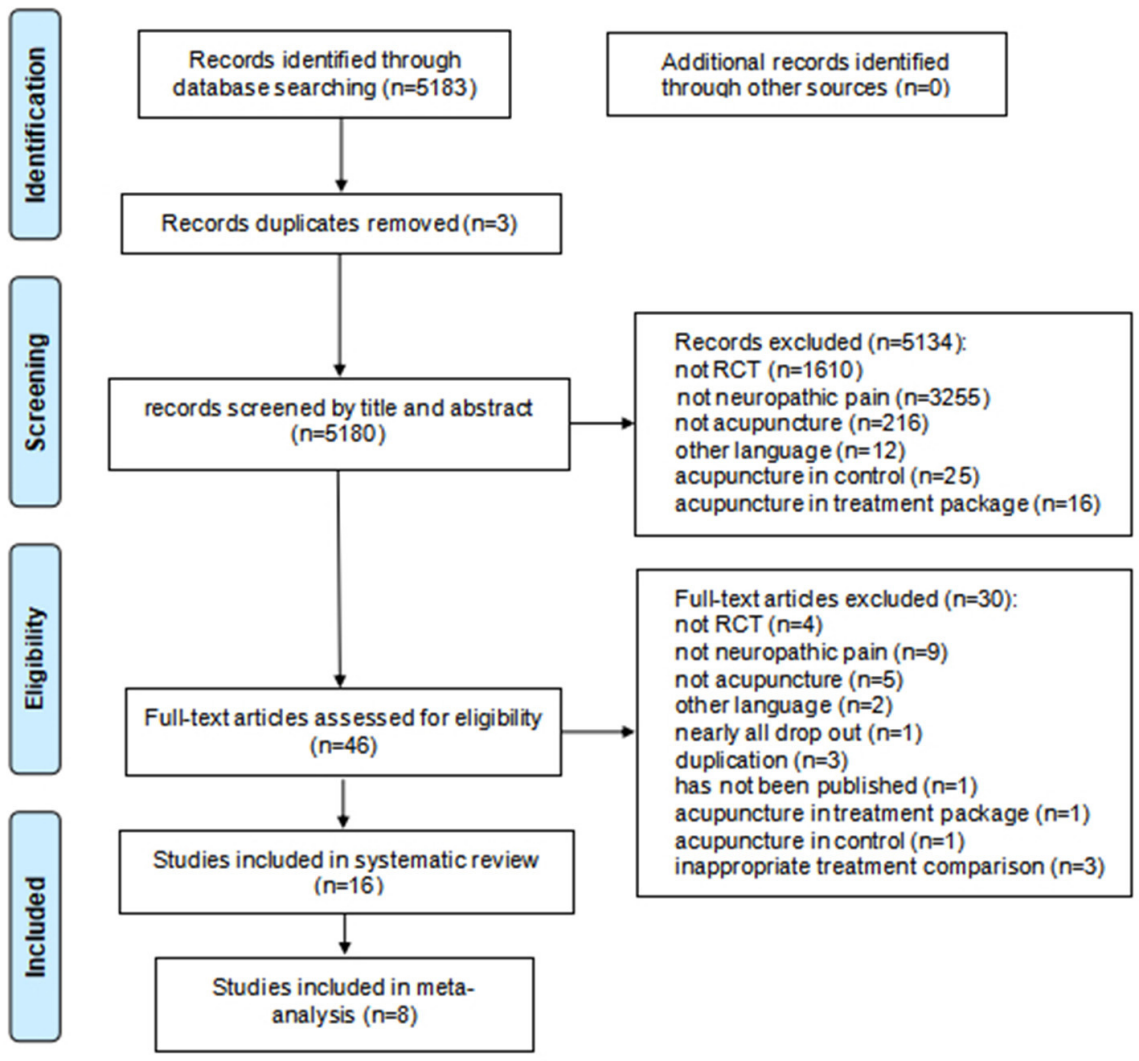
Study flow diagram.

We obtained 5,813 studies after searching the databases. In addition to three duplicates, 5,134 records were removed for irrelevant results after screening. A total of 46 full-text articles were further screened. After excluding 30 reports that did not meet the inclusion criteria, 16 studies with 1,021 patients with NP in English were included in the systematic review. Finally, eight trials with 338 participants were conducted with a meta-analysis. The selection flow of trials is shown in Figure 1.

### Description of the included studies

All the included studies were RCTs, with two (24, 26) of which were multicenter RCTs, including one study (24) located in four centers in South Korea and the other study (26) located in Iran and China. In addition, there were two studies (20, 21) from Taiwan, China, five studies (23, 27, 34–36) from the United States, three studies (19, 25, 37) from Iran, two studies (22, 38) from the United Kingdom, and one study from Croatia (39) and Italy (40), individually. The included studies contained various types of NP including one article on each of the following: postherpetic neuralgia (38), chronic sciatic pain (19), idiopathic neuropathy (40), burning mouth syndrome (39), spinal cord injury (23), and migraine (37), respectively; in addition, there were three studies on carpal tunnel syndrome (20, 21, 25) or diabetic painful neuropathy (22, 24, 40) and five studies (26, 27, 34–36) on chemotherapy-induced peripheral neuropathy. Conventional manual acupuncture was used in eight trials (20–22, 25–27, 38, 39), EA was used in four trials (19, 24, 34, 40), and auricular acupuncture was used in five trials (23, 35–38). The sessions of the interventions varied from 4 to 12 weeks, and the duration of treatments was from 20 to 30 min except for semi-permanent auricular acupuncture. A total of 10 studies (21, 23–27, 34–36, 40) reported follow-up investigations, nine (23–27, 34, 35, 40) of which ranged from 4 to 12 weeks, and one (21) was conducted for 1 year. Seven studies (20, 22, 25, 26, 34, 36, 37) mentioned the background of acupuncture practitioners, of which six (20, 22, 25, 34, 36, 37) were carried out by acupuncturists and one (26) was carried out by physicians with acupuncture experience. Moreover, 10 studies (19–27) reported positive effects, five studies (34, 36, 38–40) reported negative effects, and one study (35) did not report any clear effect on pain intensity. Detailed information is shown in Table 1.

**Table 1:** General information of included trials.

**Author**	**Age (years old)**	**Type of NP**	**Sample size (TG:CG)**	**TG/CG**	**Outcome measures**	**Follow-up**	**Registration**
Lewith et al. (38)	49–87	Post-herpetic neuralgia	62 (30:32)	AA or BA/mock transcutaneous nerve stimulation	7-point verbal pain scale	NR	NR
Hollisaz (19)	20–50	Chronic sciatic pain	119 (41:38:40)	EA/physiotherapy/placebo (SEA)	Pain reduction percent of MMPC visual scale	NR	NR
Yang et al. (20)	TG:49.30 ± 8.90; CG:49.90 ± 10.30	Carpal tunnel syndrome	77 (38:39)	A/prednisolone	GSS	NR	NR
Penza et al. (40)	43–75	Axonal polyneuropathy (4 diabetes neuropathy and 12 idiopathic neuropathy)	16 (11:5)	EA/SEA	VAS	15, 30 d	NR
Yang et al. (21)	TG:49.30 ± 8.90; CG:49.90 ± 10.30	Carpal tunnel syndrome	77 (38:39)	A/prednisolone	GSS	1 y	NCT01014221
Garrow et al. (22)	TG:68 ± 11.10; CG:63 ± 10.80	Diabetic painful neuropathy	45 (24:21)	A/SA	LANNS, VAS	NR	ISRCTN39740785
Jurisic Kvesic et al. (39)	TG:66.70 ± 12.00; CG:63.20 ± 14.00	Burning mouth syndrome	42 (20:22)	A/clonazepam	LANNS, VAS	NR	NR
Greenlee et al. (34)	TG:51.80 ± 10.70; CG:48.30 ± 12.00	Stage I-III breast cancer with CIPN	63 (31:32)	EA/SEA	BPI-SF subscale (intensity worst pain), NPS-4	4 w	NCT01163682
Estores et al. (23)	18–65	Spinal cord injury	24 (13:11)	AA/W (NI)	NRS	4 w	NR
Shin et al. (24)	>19	Painful diabetic peripheral neuropathy	126 (63:63)	EA/UC (NI)	PI-NRS, McGill pain questionnaire	4, 8 w	KCT0001135
Lu et al. (27)	>18	Stage I–III breast cancer with grade 1 or higher CIPN	40 (20:20)	A/W (UC)	BPI-SF subscales (pain severity, pain interference, average pain)	8 w	NCT02129686
Bahrami-Taghanaki et al. (25)	36.36 ± 7.74	Mild-to-moderate carpal tunnel syndrome	60 (30:30)	A/celebrex	GSS, GSS subscales (pain, numbness, tingling, muscle weakness, night awakening)	12 w	IRCT2012122811912N1
Bao et al. (35)	>18	Solid tumors with moderate to severe CIPN	75 (27:24:24)	AA + EA/SA/UC (NI)	NRS pain, NRS tingling, NRS numbness	4 w	NCT03183037
Iravani et al. (26)	TG:57.95 ± 10.39; CG:58.79 ± 8.36	Cancers with CIPN	40 (20:20)	A/vit B1 and gabapentin	NRS	4 w	IRCT20190615043900N1
Bao et al. (36)	>18	Solid tumors with moderate to severe CIPN	75 (27:24:24)	AA + EA/SA/UC (NI)	NRS	4 w	NCT03183037
Habibabadi et al. (37)	TG:37.10 ± 9.33; CG:36.65 ± 8.86	Migraine	80 (40:40)	AA/SAA	VAS	NR	IRCT20200213046477N1

TG, treatment group; CG, control group; NP, neuropathic pain; CIPN, chemotherapy-induced peripheral neuropathy; A, acupuncture; EA, electroacupuncture; AA, auricular acupuncture; SA, sham acupuncture; SEA, sham EA; SAA, sham auricular acupuncture; UC, usual care; NI, no intervention; R, rehabilitation; W, waiting, NR, not reported; GSS, global symptom score; LANSS, leeds assessment of neuropathic symptoms and signs; BPI-SF, brief pain inventory-short form; BPI, brief pain inventor; NRS, numeric rating scale; ADPS, average daily pain score; VAS, visual analog scale; PI-NRS, pain intensity numerical rating scale; y, year(s); w, week(s); d, day(s).

### Risk of bias within trials

**Figure 2:**
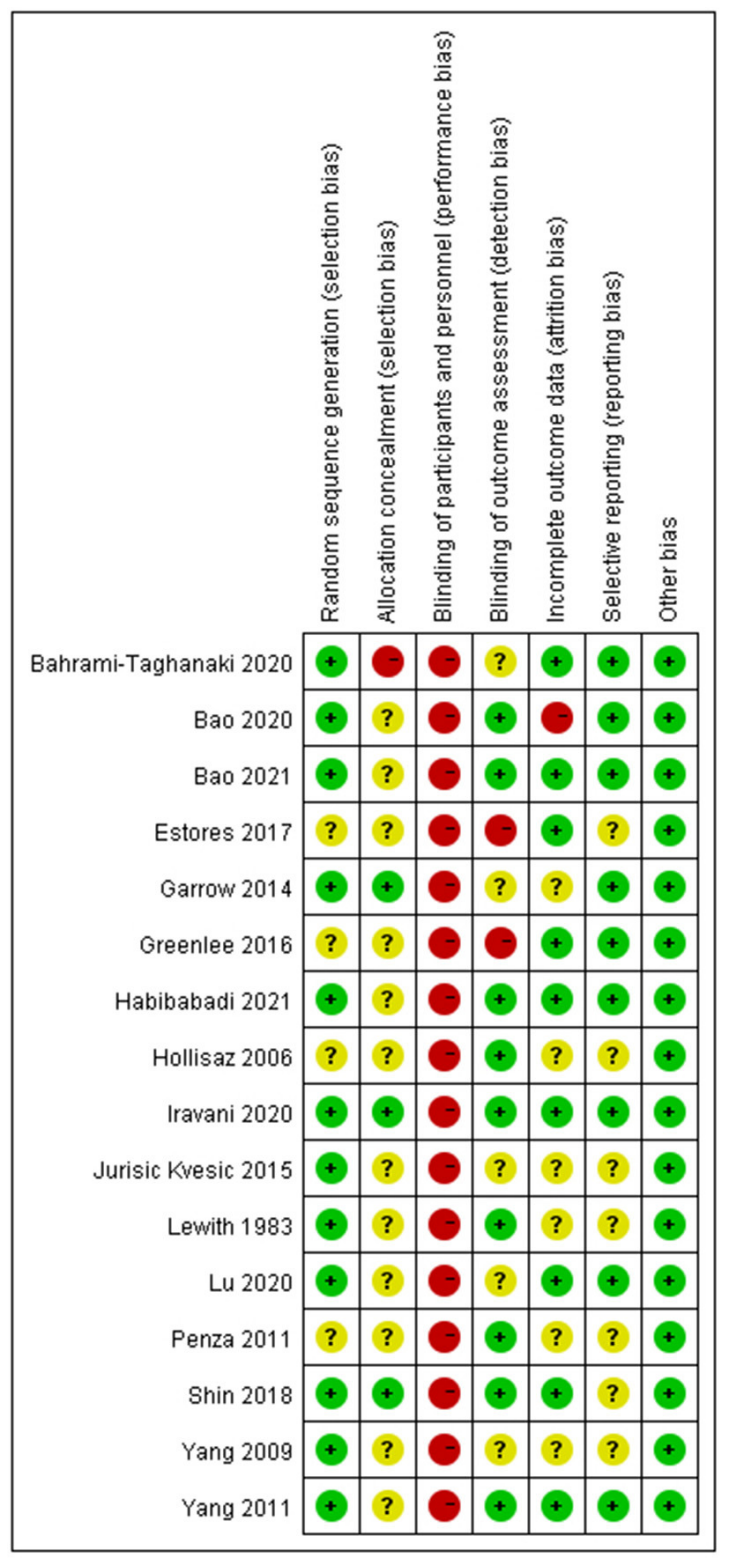
Risk of bias summary.

**Figure 3:**
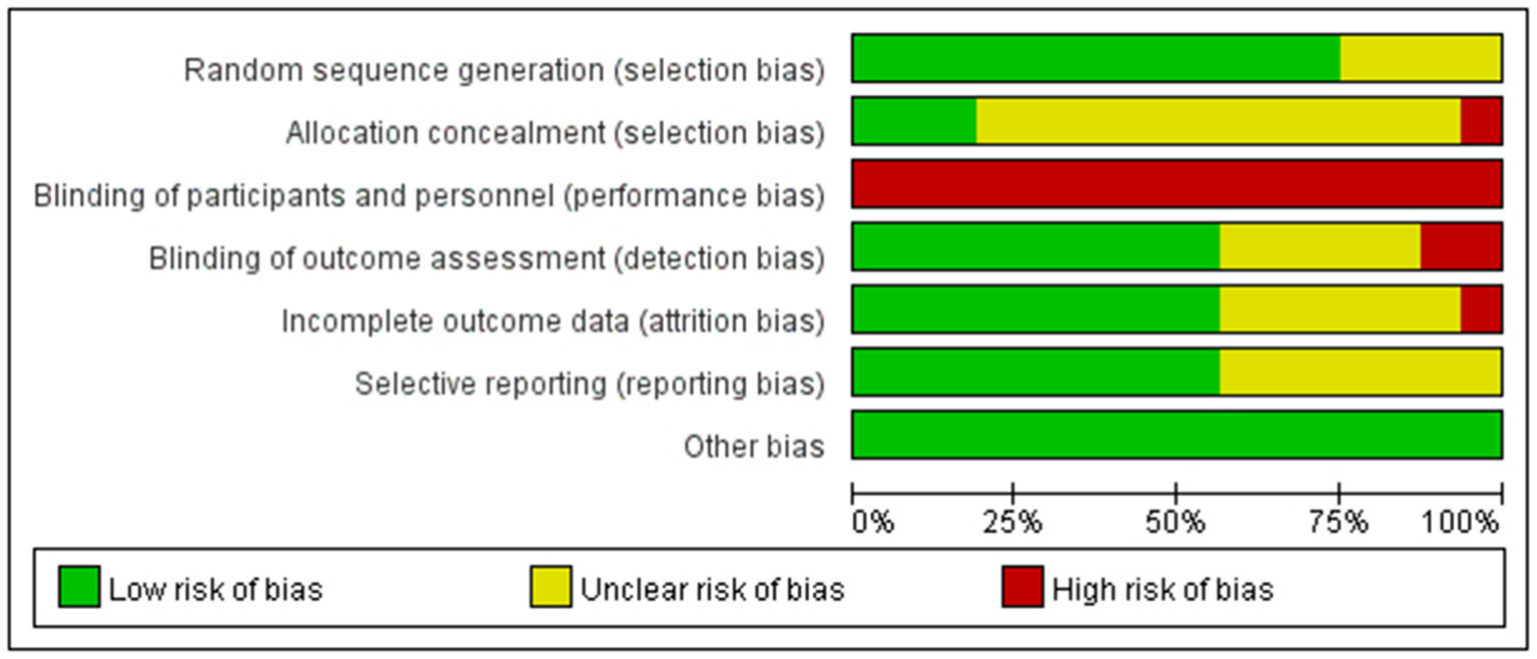
Risk of bias graph.

A total of 12 (19–22, 24–26, 35, 38, 39) of the included RCTs were evaluated with a low risk of bias of randomization sequence generation with a detailed description of randomization methods. Seven RCTs (20–22, 24, 35, 36, 38) used a computer-generated randomization list, one trial (39) used a simple randomization method of flipping a coin, one trial (25) used the random numbers table, and three trials (25, 27, 37) used a random allocation software. Four trials (19, 23, 34, 40) lack detailed information and resulted in an unclear risk of bias of randomization. Three trials (22, 24, 26), placing their information sequentially with sealed opaque envelopes, were evaluated with the low risk of bias of allocation concealment. One trial (25) used open randomization of random numbers table, which resulted in a high risk of bias of concealment. The remaining 12 trials (19–21, 23, 27, 34–40) without sufficient description in detail were regarded as unclear risk of bias of allocation concealment. None of the trials were double-blinded because the acupuncturists were not blinded. Nine trials (19, 21, 24, 26, 35–38, 40) were single-blinded in outcome assessment, five trials (20, 22, 25, 27, 39) did not describe methods of blinding, and two trials (23, 34) indicated that assessors were not blinded. Three trials (25, 26, 37) reported no attrition in follow-up studies, while seven trials (21–24, 27, 34, 36) have intention-to-treat (ITT) analysis. However, one trial (35) did not address the loss of follow-up treatments, while five trials (19, 20, 38–40) had no description of the loss of follow-up. Nine trials (20, 22, 25–27, 34–36) registered online previously with certain outcomes and led to a low risk of bias in selective reporting. One trial (24) did not report the complete outcome data. Therefore, we were unable to extract the data for meta-analysis, resulting in the unclear risk of bias. The left trials (19, 21, 23, 38–40) had an unclear risk of bias without detailed reports. In other sources of bias, all trials were evaluated with low risk. The brief information is shown in Figures 2, 3.

### Effects of interventions

#### Primary outcome (changes in pain intensity)

**Figure 4:**
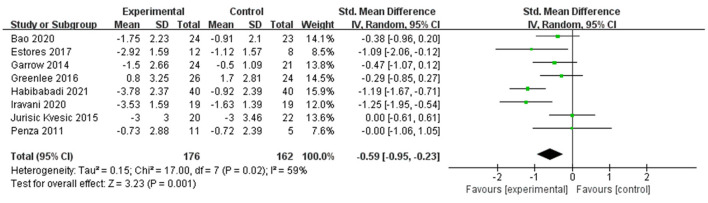
Forest plot and meta-analysis of changes in pain intensity.

Changes in pain intensity (including changes in VAS, NRS, and BPI-SF worst pain score) occurred in eight RCTs (22, 23, 26, 34, 35, 37, 39, 40) with 338 participants. They investigated the effect of acupuncture on changes in pain intensity, including four trials (22, 26, 39, 40) on conventional manual acupuncture, three trials (23, 35, 37) on auricular acupuncture, and one trial (34) on EA. Using a random effect model among the results (*P* = 0.02, *I*^2^ = 59%), a significant effect was shown in changes in pain intensity in the acupuncture group (SMD −0.59, 95% CI: −0.95 to −0.23, *P* = 0.001) (Figure 4). Eight trials (19–21, 24, 27, 35, 36, 38) were not pooled in the meta-analysis because one trial (24) did not report complete data and seven trials (19–21, 27, 35, 36, 38) did not report the relative outcome.

#### Subgroup analysis

**Figure 5:**
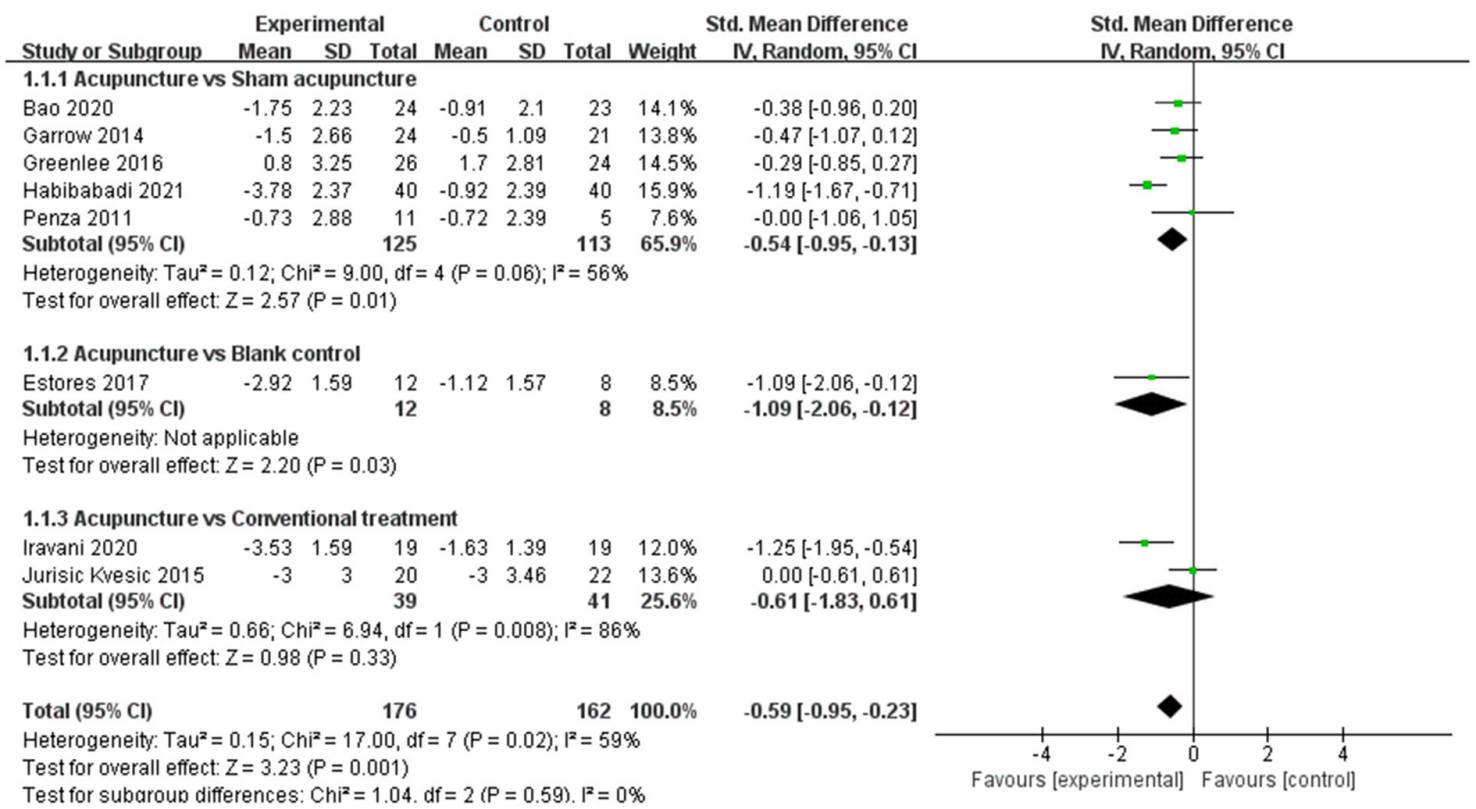
Subgroup analysis of changes in pain intensity.

Subgroup analysis was used to verify if different interventions of the control group would affect the changes in pain intensity. Five trials (22, 34, 35, 37, 40) including 238 patients using a random effect model indicated that acupuncture was more effective in improving changes in pain intensity than sham acupuncture (SMD −0.54, 95% CI: −0.95 to −0.13, *P* = 0.01). Two trials (26, 39) evaluated the effect on changes in pain intensity with a random effect model among 80 patients in the comparison of acupuncture and conventional treatments including clonazepam in the study of Jurisic Kvesic et al. (39) and vitamin B1 and gabapentin in the study of Iravani et al. (26), and there was no significant difference (SMD −0.61, 95% CI: −1.83 to 0.61, *P* = 0.33). One trial (23) compared acupuncture with blank control evaluating the effect on changes in pain intensity with a significant difference (Figure 5).

According to the subgroup analysis, the acupuncture group had higher effectiveness than sham intervention or blank control for changes in pain intensity, but there is no significant difference between acupuncture and conventional treatments.

#### Sensitivity analysis and publication bias

**Figure 6:**
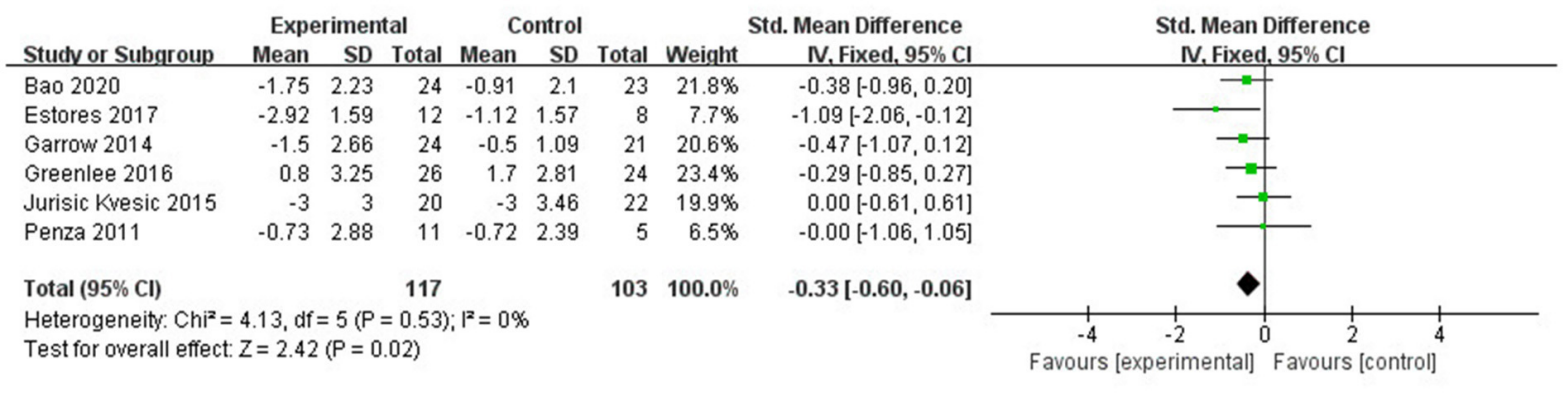
Sensitivity analysis: Forest plot and meta-analysis of changes in pain intensity after removing the studies of Iravani et al. (26) and Habibabadi et al. (37).

**Figure 7:**
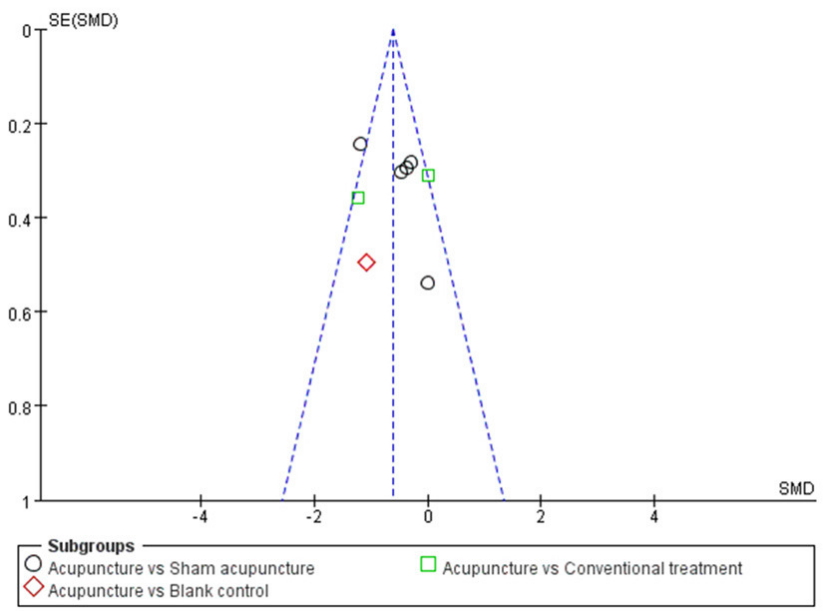
Funnel plots of changes in pain intensity.

The sensitivity analysis showed that studies of Iravani et al. (26) and Habibabadi et al. (37) may be the main cause of heterogeneity as *I*^2^ dropped to 0% after they were removed (Figure 6). The funnel plot of changes in pain intensity was symmetric, which means no publication bias was detected (Figure 7).

### Quality assessment

We evaluated the available evidence with the GRADE tool; the quality of evidence on acupuncture for NP was graded as “very low.” Details are shown in Table 2.

**Table 2:** Effect of acupuncture on changes in pain intensity in patients with neuropathic pain.

**Certainty assessment**							**No. of patients**		**Effect**		**Certainty**	**Importance**
**No. of studies**	**Study design**	**Risk of bias**	**Inconsistency**	**Indirectness**	**Imprecision**	**Other considerations**	**Acupuncture**	**Sham acupuncture or blank control or conventional treatments**	**Relative** **(95% CI)**	**Absolute** **(95% CI)**		
**New outcome**												
8	Randomized trials	Serious^a^	Serious^b^	Not serious	Serious^c^	None	176	162	–	SMD **0.59 SD lower** (0.95 lower to 0.23 lower)	⊕○○○ Very low	CRITICAL

CI, confidence interval; SMD, standardized mean difference.

^a^High risk of bias due to the lack of allocation concealment, blinding, and incomplete outcome data.

^b^Moderate heterogeneity.

^c^Wide CI.

### Adverse events

A total of 11 RCTs (20–22, 24, 26, 27, 34–36, 39, 40) reported safety conditions, while five RCTs (19, 23, 25, 37, 38) did not mention AE. Two RCTs (21, 40) reported that there was no AE, especially one RCT (21) reported that there was no AE during a 1-year follow-up. Three RCTs (24, 26, 39) reported that there was no AE associated with acupuncture treatments. AEs induced by acupuncture mentioned above involved pain, discomfort, paresthesia, minor swelling, bruising and ecchymosis of the acupoint sites, which were mild and reversible (20, 22, 27, 34, 36). Moreover, AEs in the control group were also reported, including nausea and epigastralgia caused by prednisolone (20); drowsiness, dizziness, and nausea induced by clonazepam (39); and somnolence and dizziness caused by vitamin B1 and gabapentin (26), which were mainly induced by side effects of conventional treatment for NP. Details are shown in Table 3.

**Table 3:** Characteristic of included trials cont.

**Author**	**Acupoints**	**Session**	**Effect**	**Adverse events**
Lewith et al. (38)	AA was used first with most tender point on the pinna, but if no improvement occurred after 2 or 3 treatments it was changed to BA with acupoints on the site and distribution of the pain.	10 min, 8 times, 8 w	Negative	NR
Hollisaz (19)	NR	EA or placebo 20 min; physiotherapy 30 min, 15 times, 4w	Positive	NR
Yang et al. (20)	PC-7, PC-6	A 30 min, 8 times, 4 w; prednisolone 20 mg qd 2 w	Positive	No serious adverse effects were noted. 5% patients in TG: local pain, ecchymosis and paresthesia; 18% patients in CG: nausea and epigastralgia
Penza et al. (40)	ST36, SP6, LR3, and BL60	30 min, 6 times, 6 w	Negative	No side effect was recorded
Yang et al. (21)	PC-7, PC-6	A 30 min, 8 times, 4 w; prednisolone 20 mg qd, 2 w	Positive	No long-term AEs
Garrow et al. (22)	LR3, KI3, SP6, SP10, and ST36	30 min, 10 times, 10 w	Positive	One chest pain, one leg pain in TG; one localized swelling of leg in CG
Jurisic Kvesic et al. (39)	ST8, GB2, TE21, SI19, SI18, LI4, and GV20	A 30 min, 12 times, 4 w; clonazepam 0.5 mg qd first 2 w and 0.5 mg bid further 2 w	Negative	Five drowsiness, dizziness, and nausea in CG
Greenlee et al. (34)	GB34, ST 36, LI4, LI10, L3, L5, C5, and C7	30 min, 12 times, 12 w	Negative	One needle site with grade one discomfort, minor swelling, and bruising in TG
Estores et al. (23)	Auricular acupuncture: anterior cingulate, thalamus, omega-2, Shen Men, and point zero	Semi-permanent, 8 times, 8 w	Positive	NR
Shin et al. (24)	ST36, GB39, SP9, SP6, LR3, GB41, and Bafeng (EX-LE10)	30 min, 16 times, 8 w	Positive	24 AEs and six serious AEs. None of them related to TG
Lu et al. (27)	LI-11, TW-5, SP-9, ST-36, SP-6, K-3, LR-3, Yin Tang, Baxie, and Qiduan (1st−5th)	30 min, 18 times, 8 w	Positive	One grade one pruritis in feet, one grade two joint pain possibly related to the acupuncture
Bahrami-Taghanaki et al. (25)	LI-11, TB-5, PC-8, LI-4, PC-7, SI-3, TB-4, and ST-36	30 min, 12 times, 4 w; celebrex 100 mg, tid, 4 w	Positive	NR
Bao et al. (35)	Auricular acupuncture: Shen Men, point zero, and a third electrodermal active point; body acupuncture: LI-4, PC-6, SI-3, LR-3, GB-42, ST-40, Bafeng 2, and Bafeng 3	20 min, 10times, 8 w	Unclear	AEs were few and mild
Iravani et al. (26)	CV 6, GV 20, ST 36, SP 6, LI 4, LI 11, LR 3, Baxie (EX-UE 9), and Bafeng (EX-LE 10)	20 min, 12 sessions, 4 w; vit B1 300 mg and gabapentin 900 mg, qd, 4 w	Positive	One somnolence and dizziness in CG
Bao et al. (36)	Auricular acupuncture: Shen Men, point zero, and a third electrodermal active point, and bilateral; body acupuncture: LI-4, PC-6, SI-3, LR-3, GB-43, ST-40, Bafeng 2, and Bafeng 3	30 min, 10 times, 8 w	Negative	Three grade one pain at the needling site, two bruising, one feel claustrophobic with the eye mask on in TG
Habibabadi et al. (37)	Auricular acupuncture: Sympathetic, Gallbladder, GB3, GB40, Lesser Occipital Nerve, Thalamus, Ear Apex, Forehead, Zero, Shen Men, PGE1, PGE2, Liver, Hypothalamus, Frustration, Temple, Occiput, local cervical point (back), local cervical point (front), and worry point	Semi-permanent, 2 times, 4 w	Positive	NR

TG, treatment group; CG, control group; NR, not reported; AEs, adverse events; EA, electroacupuncture.

## Discussion

This study included a systematic review of 16 RCTs with 1,021 patients and a meta-analysis of eight studies with 338 subjects assessing the effect on pain intensity and safety of acupuncture in patients with NP. Our findings from the qualitative analysis of the systematic review showed an unclear effect of acupuncture on improving pain intensity in patients with NP because 10 studies (19–27) reported positive effects, five studies (34, 36, 38–40) reported negative effects, and one study (35) did not report any clear effect on pain intensity. However, the results of the meta-analysis indicated that acupuncture was an effective intervention for patients with NP. In addition, 11 trials (20–22, 24, 26, 27, 34–36, 39, 40) reported safety conditions, and acupuncture-induced AEs were mild and reversible, indicating that acupuncture is a relatively safe intervention for patients with NP.

Sham acupuncture and blank control are conventionally designed to help reduce bias in assessing the specific effect of acupuncture. According to the results of the subgroup analysis, acupuncture had higher effectiveness than sham acupuncture or blank control for changes in pain intensity. This means that acupuncture is also an effective treatment for NP. However, there was no significant difference between acupuncture and conventional treatments for NP. Nevertheless, it is notable that compared with the side effects of conventional treatments, acupuncture-induced AEs were mild and reversible. Therefore, patients who respond to the limited effects of conventional treatments or feel difficult to withstand the side effects of conventional treatments may consider acupuncture as an alternative. In brief, acupuncture may be beneficial to improve the pain intensity of patients with NP in a relatively safe means, and as a complementary part to provide more specific evidence to improve clinical practice. The results of the sensitivity analysis and funnel plot showed that the effect of acupuncture on the changes in pain intensity in patients with NP was robust. Furthermore, the “very low” GRADE results of changes in pain intensity may suggest this treatment to clinical practice with a recommendation level of “very low.” Ultimately, the interpretation of our results should be performed cautiously due to the low methodological quality of selected publications.

Developing after nerve injuries, NP occurs in deleterious changes in damaged neurons and goes along with the nociceptive and descending modulatory pathways of the central nervous system (41). Sensitization of nociceptive pathways are mainly based on maladaptive structural alterations, cell interactions, and molecular signaling, including changes in the activation of immune cells, glial-derived mediators, ion channels, and epigenetic regulation (42). Ali et al. (43) found that EA can improve NP by stimulating the spinal microglial expression of IL-10 and subsequent β-endorphin. Liu et al. (44) indicated that EA can modulate miR-214 to suppress neuronal apoptosis by targeting Bax and inhibiting the expression of the Nav1.3 channel. Jang et al. (45) suggested that acupuncture can ameliorate chronic NP-induced comorbid conditions by changing the DNA methylation of Nr4a1, Rasgrp1, Rassf1, and Chkb in the PFC. Chen et al. (46) suggested that EA can ameliorate tactile allodynia after peripheral nerve injury by suppressing the excessive expression of IFN-γ in the spinal cord and subsequent P2X4R. In addition, several studies indicated that EA can relieve NP by suppressing PKC-dependent membrane P2X3 upregulation in DRG (47–49). Lee et al. (50) found that acupuncture can relieve pain by inhibiting JNK activation in astrocytes after SCI. Li et al. (51) suggested that EA can improve paclitaxel-induced peripheral NP by suppressing TLR4 signaling and TRPV1 upregulation in DRG neurons, which can further result in reduced spinal glial activation. Moreover, several studies found that opioid receptors or spinal muscarinic receptors can significantly suppress mechanical allodynia with NP (52–54). In addition, Napadow et al. (55) indicated that patients with carpal tunnel syndrome evaluated by fMRI respond to acupuncture through a coordinated limbic network including the hypothalamus and amygdala. Currently, the mechanism of acupuncture for NP has not met an agreement, and thus more concentration is needed to focus on how acupuncture relieves NP.

### Study strengths and limitations

Our study has multiple strengths. First, our review focused on the effect of using acupuncture alone, so we excluded the studies of mixed therapies and conducted a subgroup study of sham acupuncture or blank control in the control group to verify whether acupuncture is effective for NP. Second, a previously published meta-analysis (30) showed that it is challenging to either support or refute the effect of acupuncture on NP. Nevertheless, the study only included two manual acupuncture RCTs (22, 56) on pain intensity in its meta-analysis. In contrast, we include more RCTs with a larger sample size and more acupuncture manipulations. Moreover, compared with the previous study (30), patients with central NP and peripheral NP were included in our study, which may more strongly support the hypothesis that acupuncture on NP is effective. Third, many studies were performed at multiple locations and in different countries, covering a more ethnically and culturally diverse sample, which may reduce selection bias and improve external validity. Fourth, sensitivity analysis and funnel plot were conducted, demonstrating that the meta-analysis was stable and irreversible without publication bias. Fifth, most of the studies were longitudinal and one of them was followed for 1 year. To some extent, our study provided supporting evidence for the clinical practice of acupuncture in the treatment of patients with NP.

However, there were also some limitations to this study. First, in the qualitative analysis of the systematic review, six (34–36, 38–40) of the 16 trials indicated a negative or ambiguous effect on pain intensity of acupuncture for NP. Second, the outcomes of life quality evaluation were inadequate to pool to perform the meta-analysis. Third, GRADE was rated as “very low.” The quality of total studies was low, especially in the area of allocation concealment and participant and personnel blindness.

### Implication for further research

More high-quality studies on acupuncture for patients with NP are needed to enlarge the sample size and reduce bias. Longer follow-up trials are required to observe the long-term effect of acupuncture in the treatment of NP. Consolidated Standards of Reporting Trial (CONSORT) statement and STRICTA checklists (57, 58) should be followed in future studies. To achieve double-blinding, standardized trial design, a timely data storage system and a well-coordinated team are needed to help performed sham intervention successfully, which can refer to pragmatic-explanatory continuum indicator summary (PRECIS) or PRECIS-2 (59–61).

## Conclusion

The acupuncture group had higher effectiveness than sham intervention or blank control for changes in pain intensity, but there is no significant difference between acupuncture and conventional treatments in treating NP. The acupuncture-induced adverse events were mild and reversible. However, the interpretation of our results should be performed cautiously due to the low methodological quality of selected publications.

## Data availability statement

The raw data supporting the conclusions of this article will be made available by the authors, without undue reservation.

## Author contributions

MX conceptualized and designed the study. ZF and SC contributed to drafting the text and the analysis of data. JW searched and screened the data. HY, YW, LL, ZY, BH, and HZ identified relevant articles and extracted data. XZ analyzed data. All authors approved the final manuscript.

## References

[1] Colloca L Ludman T Bouhassira D Baron R Dickenson AH Yarnitsky D . Neuropathic pain. Nat Rev Dis Primers. (2017) 3:17002. 10.1038/nrdp.2017.2 28205574 PMC5371025

[2] Schlereth T. Guideline “diagnosis and non-interventional therapy of neuropathic pain” of the German society of neurology (Deutsche Gesellschaft Fur Neurologie). Neurol Res Pract. (2020) 2:16. 10.1186/s42466-020-00063-3 33324922 PMC7650069

[3] DiBonaventura MD Sadosky A Concialdi K Hopps M Kudel I Parsons B . The prevalence of probable neuropathic pain in the US: results from a multimodal general-population health survey. J Pain Res. (2017) 10:2525–38. 10.2147/JPR.S127014 29138590 PMC5677393

[4] Schmader KE Baron R Haanpaa ML Mayer J O'Connor AB Rice AS . Treatment considerations for elderly and frail patients with neuropathic pain. Mayo Clin Proc. (2010) 85:S26–32. 10.4065/mcp.2009.0646 20194145 PMC2844008

[5] Inoue S Taguchi T Yamashita T Nakamura M Ushida T. The prevalence and impact of chronic neuropathic pain on daily and social life: a nationwide study in a Japanese population. Eur J Pain. (2017) 21:727–37. 10.1002/ejp.977 28107599 PMC5363338

[6] Li KL Chen YM Wang XQ Hu HY. Bibliometric analysis of studies on neuropathic pain associated with depression or anxiety published from 2000 to 2020. Front Hum Neurosci. (2021) 15:729587. 10.3389/fnhum.2021.729587 34552477 PMC8450598

[7] Guntel M Huzmeli ED Melek I. Patients with neuropathic pain have poor sleep quality. J Nerv Ment Dis. (2021) 209:505–9. 10.1097/NMD.0000000000001325 34170859

[8] Daniel HC Narewska J Serpell M Hoggart B Johnson R Rice AS. Comparison of psychological and physical function in neuropathic pain and nociceptive pain: implications for cognitive behavioral pain management programs. Eur J Pain. (2008) 12:731–41. 10.1016/j.ejpain.2007.11.006 18164225

[9] Melikoglu MA Celik A. Does neuropathic pain affect the quality of sleep? Eurasian J Med. (2017) 49:40–3. 10.5152/eurasianjmed.2017.16261 28416931 PMC5389492

[10] McCarberg BH Billington R. Consequences of neuropathic pain quality-of-life issues and associated costs. Am J Manag Care. (2006) 12(9 Suppl):S263–8. 16774458

[11] Schaefer C Sadosky A Mann R Daniel S Parsons B Tuchman M . Pain severity and the economic burden of neuropathic pain in the United States: beat neuropathic pain observational study. Clinicoecon Outcomes Res. (2014) 6:483–96. 10.2147/CEOR.S63323 25378940 PMC4218900

[12] Bannister K Sachau J Baron R Dickenson AH. Neuropathic pain: mechanism-based therapeutics. Annu Rev Pharmacol Toxicol. (2020) 60:257–74. 10.1146/annurev-pharmtox-010818-021524 31914896

[13] Serrano Afonso A Carnaval T Videla Cés S. Combination therapy for neuropathic pain: a review of recent evidence. J Clin Med. (2021) 10:533. 10.20944/preprints202106.0200.v2 34441829 PMC8396869

[14] Selvy M Cuménal M Kerckhove N Courteix C Busserolles J Balayssac D. The safety of medications used to treat peripheral neuropathic pain, part 1 (antidepressants and antiepileptics): review of double-blind, placebo-controlled, randomized clinical trials. Exp Opin Drug Saf. (2020) 19:707–33. 10.1080/14740338.2020.1764934 32363948

[15] Sachau J Baron R. Neuropathic pain therapy: a puzzle of different approaches to stratify patients. Pain. (2021) 162:993–4. 10.1097/j.pain.0000000000002120 33086285

[16] Knezevic NN Cicmil N Knezevic I Candido KD. Discontinued neuropathic pain therapy between 2009 and 2015. Exp Opin Investig Drugs. (2015) 24:1631–46. 10.1517/13543784.2015.1099627 26472477

[17] Rowin J. Integrative neuromuscular medicine: neuropathy and neuropathic pain: consider the alternatives. Muscle Nerve. (2019) 60:124–36. 10.1002/mus.26510 31074875

[18] Musial F. Acupuncture for the treatment of pain: a mega-placebo? Front Neurosci. (2019) 13:1110. 10.3389/fnins.2019.01110 31680841 PMC6811493

[19] Hollisaz M. Use of electroacupuncture for treatment of chronic sciatic pain. Internet J Pain Symptom Control Palliat Care. (2007) 5:1–4. 10.5580/993

[20] Yang CP Hsieh CL Wang NH Li TC Hwang KL Yu SC . Acupuncture in patients with carpal tunnel syndrome: a randomized controlled trial. Clin J Pain. (2009) 25:327–33. 10.1097/AJP.0b013e318190511c 19590482

[21] Yang C Wang N Li T Hsieh C Chang H Hwang K . A randomized clinical trial of acupuncture vs. oral steroids for carpal tunnel syndrome: a long-term follow-up. J Pain. (2011) 12:272–9. 10.1016/j.jpain.2010.09.001 21111685

[22] Garrow AP Xing M Vere J Verrall B Wang L Jude EB. Role of acupuncture in the management of diabetic painful neuropathy (Dpn): a pilot rct. Acupunct Med. (2014) 32:242–9. 10.1136/acupmed-2013-010495 24657491

[23] Estores I Chen K Jackson B Lao L Gorman PH. Auricular acupuncture for spinal cord injury related neuropathic pain: a pilot controlled clinical trial. J Spinal Cord Med. (2017) 40:432–8. 10.1080/10790268.2016.1141489 26869339 PMC5537960

[24] Shin KM Lee S Lee EY Kim CH Kang JW Lee CK . Electroacupuncture for painful diabetic peripheral neuropathy: a multicenter, randomized, assessor-blinded, controlled trial. Diabetes Care. (2018) 41:e141–e2. 10.2337/dc18-1254 30061320

[25] Bahrami-Taghanaki H Azizi H Hasanabadi H Jokar MH Iranmanesh A Khorsand-Vakilzadeh A . Acupuncture for carpal tunnel syndrome: a randomized controlled trial studying changes in clinical symptoms and electrodiagnostic tests. Altern Ther Health Med. (2020) 26:10–6. 31634868

[26] Iravani S Kazemi Motlagh AH Emami Razavi SZ Shahi F Wang J Hou L . Effectiveness of acupuncture treatment on chemotherapy-induced peripheral neuropathy: a pilot, randomized, assessor-blinded, controlled trial. Pain Res Manag. (2020) 2020:2504674. 10.1155/2020/2504674 32676134 PMC7341378

[27] Lu W Giobbie-Hurder A Freedman RA Shin IH Lin NU Partridge AH . Acupuncture for chemotherapy-induced peripheral neuropathy in breast cancer survivors: a randomized controlled pilot trial. Oncologist. (2020) 25:310–8. 10.1634/theoncologist.2019-0489 32297442 PMC7160396

[28] Bäumler P Zhang W Stübinger T Irnich D. Acupuncture-related adverse events: systematic review and meta-analyses of prospective clinical studies. BMJ Open. (2021) 11:e045961. 10.1136/bmjopen-2020-045961 34489268 PMC8422480

[29] McGeeney BE. Acupuncture is all placebo and here is why. Headache. (2015) 55:465–9. 10.1111/head.12524 25660556

[30] Ju ZY Wang K Cui HS Yao Y Liu SM Zhou J . Acupuncture for neuropathic pain in adults. Cochrane Database Syst Rev. (2017) 12:Cd012057. 10.1002/14651858.CD012057 29197180 PMC6486266

[31] Page MJ McKenzie JE Bossuyt PM Boutron I Hoffmann TC Mulrow CD . The prisma 2020 statement: an updated guideline for reporting systematic reviews. Int J Surg. (2021) 88:105906. 10.1016/j.ijsu.2021.105906 33789826

[32] JPT. H, S. G. Cochrane handbook for systematic reviews of interventions. Version 5.1.0. Cochrane Collabor. (2011) 91:25–32. Available online at: https://training.cochrane.org/handbook/archive/v5.1/

[33] Hjermstad MJ Fayers PM Haugen DF Caraceni A Hanks GW Loge JH . Studies comparing numerical rating scales, verbal rating scales, and visual analogue scales for assessment of pain intensity in adults: a systematic literature review. J Pain Symptom Manage. (2011) 41:1073–93. 10.1016/j.jpainsymman.2010.08.016 21621130

[34] Greenlee H Crew KD Capodice J Awad D Buono D Shi Z . Randomized sham-controlled pilot trial of weekly electro-acupuncture for the prevention of taxane-induced peripheral neuropathy in women with early stage breast cancer. Breast Cancer Res Treat. (2016) 156:453–64. 10.1007/s10549-016-3759-2 27013473 PMC4924571

[35] Bao T Patil S Chen C Zhi IW Li QS Piulson L . Effect of acupuncture vs. sham procedure on chemotherapy-induced peripheral neuropathy symptoms: a randomized clinical trial. JAMA Netw Open. (2020) 3:e200681. 10.1001/jamanetworkopen.2020.0681 32159808 PMC7066475

[36] Bao T Baser R Chen C Weitzman M Zhang YL Seluzicki C . Health-related quality of life in cancer survivors with chemotherapy-induced peripheral neuropathy: a randomized clinical trial. Oncologist. (2021) 26:e2070–e8. 10.1002/onco.13933 34390283 PMC8571772

[37] Habibabadi MR Ashtari F Raeisi I. Effect of auricular acupuncture with semi-permanent ear needles on controlling migraine symptoms: a single-blind randomized clinical trial. J Acupunct Meridian Stud. (2021) 14:58–66. 10.51507/j.jams.2021.14.2.58 35770540

[38] Lewith GT Field J Machin D. Acupuncture compared with placebo in post-herpetic pain. Pain. (1983) 17:361–8. 10.1016/0304-3959(83)90167-7 6664681

[39] Jurisic Kvesic A Zavoreo I Basic Kes V Vucicevic Boras V Ciliga D Gabric D . The effectiveness of acupuncture vs. clonazepam in patients with burning mouth syndrome. Acupunct Med. (2015) 33:289–92. 10.1136/acupmed-2015-010759 25987645

[40] Penza P Bricchi M Scola A Campanella A Lauria G. Electroacupuncture is not effective in chronic painful neuropathies. Pain Med. (2011) 12:1819–23. 10.1111/j.1526-4637.2011.01230.x 21917117

[41] Cohen SP Mao J. Neuropathic pain: mechanisms and their clinical implications. BMJ. (2014) 348:f7656. 10.1136/bmj.f7656 24500412

[42] Finnerup NB Kuner R Jensen TS. Neuropathic pain: from mechanisms to treatment. Physiol Rev. (2021) 101:259–301. 10.1152/physrev.00045.2019 32584191

[43] Ali U Apryani E Wu HY Mao XF Liu H Wang YX. Low frequency electroacupuncture alleviates neuropathic pain by activation of spinal microglial Il-10/?-endorphin pathway. Biomed Pharmacother. (2020) 125:109898. 10.1016/j.biopha.2020.109898 32004977

[44] Liu J Wu Y. Electro-acupuncture-modulated Mir-214 prevents neuronal apoptosis by targeting bax and inhibits sodium channel Nav13 expression in rats after spinal cord injury. Biomed Pharmacother. (2017) 89:1125–35. 10.1016/j.biopha.2017.02.077 28298073

[45] Jang JH Song EM Do YH Ahn S Oh JY Hwang TY . Acupuncture alleviates chronic pain and comorbid conditions in a mouse model of neuropathic pain: the involvement of DNA methylation in the prefrontal cortex. Pain. (2021) 162:514–30. 10.1097/j.pain.0000000000002031 32796318 PMC7808350

[46] Chen XM Xu J Song JG Zheng BJ Wang XR. Electroacupuncture inhibits excessive interferon-Γ evoked up-regulation of P2x4 receptor in spinal microglia in a Cci rat model for neuropathic pain. Br J Anaesth. (2015) 114:150–7. 10.1093/bja/aeu199 25074385

[47] Tu WZ Cheng RD Cheng B Lu J Cao F Lin HY . Analgesic effect of electroacupuncture on chronic neuropathic pain mediated by P2x3 receptors in rat dorsal root ganglion neurons. Neurochem Int. (2012) 60:379–86. 10.1016/j.neuint.2012.01.006 22269805

[48] Wang WS Tu WZ Cheng RD He R Ruan LH Zhang L . Electroacupuncture and a-317491 depress the transmission of pain on primary afferent mediated by the P2x3 receptor in rats with chronic neuropathic pain states. J Neurosci Res. (2014) 92:1703–13. 10.1002/jnr.23451 25041872

[49] Zhou YF Ying XM He XF Shou SY Wei JJ Tai ZX . Suppressing Pkc-dependent membrane P2x3 receptor upregulation in dorsal root ganglia mediated electroacupuncture analgesia in rat painful diabetic neuropathy. Purinerg Signal. (2018) 14:359–69. 10.1007/s11302-018-9617-4 30084084 PMC6298917

[50] Lee JY Choi DC Oh TH Yune TY. Analgesic effect of acupuncture is mediated *via* inhibition of Jnk activation in astrocytes after spinal cord injury. PLoS ONE. (2013) 8:e73948. 10.1371/journal.pone.0073948 24040124 PMC3767587

[51] Li Y Yin C Li X Liu B Wang J Zheng X . Electroacupuncture alleviates paclitaxel-induced peripheral neuropathic pain in rats *via* suppressing Tlr4 signaling and Trpv1 upregulation in sensory neurons. Int J Mol Sci. (2019) 20:5917. 10.3390/ijms20235917 31775332 PMC6929119

[52] Meng X Zhang Y Li A Xin J Lao L Ren K . The effects of opioid receptor antagonists on electroacupuncture-produced anti-allodynia/hyperalgesia in rats with paclitaxel-evoked peripheral neuropathy. Brain Res. (2011) 1414:58–65. 10.1016/j.brainres.2011.08.004 21872220 PMC3176966

[53] Kim JH Min BI Na HS Park DS. Relieving effects of electroacupuncture on mechanical allodynia in neuropathic pain model of inferior caudal trunk injury in rat: mediation by spinal opioid receptors. Brain Res. (2004) 998:230–6. 10.1016/j.brainres.2003.11.045 14751594

[54] Park JH Kim SK Kim HN Sun B Koo S Choi SM . Spinal cholinergic mechanism of the relieving effects of electroacupuncture on cold and warm allodynia in a rat model of neuropathic pain. J Physiol Sci. (2009) 59:291–8. 10.1007/s12576-009-0035-9 19343482 PMC10717390

[55] Napadow V Kettner N Liu J Li M Kwong KK Vangel M . Hypothalamus and amygdala response to acupuncture stimuli in carpal tunnel syndrome. Pain. (2007) 130:254–66. 10.1016/j.pain.2006.12.003 17240066 PMC1997288

[56] Han X Wang L Shi H Zheng G He J Wu W . Acupuncture combined with methylcobalamin for the treatment of chemotherapy-induced peripheral neuropathy in patients with multiple myeloma. BMC Cancer. (2017) 17:40. 10.1186/s12885-016-3037-z 28068938 PMC5223334

[57] MacPherson H Altman DG Hammerschlag R Youping L Taixiang W White A . Revised standards for reporting interventions in clinical trials of acupuncture (stricta): extending the consort statement. PLoS Med. (2010) 7:e1000261. 10.1371/journal.pmed.1000261 20543992 PMC2882429

[58] Moher D Hopewell S Schulz KF Montori V Gøtzsche PC Devereaux PJ . Consort 2010 explanation and elaboration: updated guidelines for reporting parallel group randomised trials. BMJ. (2010) 340:c869. 10.1136/bmj.c869 20332511 PMC2844943

[59] Riddle DL Johnson RE Jensen MP Keefe FJ Kroenke K Bair MJ . The pragmatic-explanatory continuum indicator summary (precis) instrument was useful for refining a randomized trial design: experiences from an investigative team. J Clin Epidemiol. (2010) 63:1271–5. 10.1016/j.jclinepi.2010.03.006 20670911 PMC2943562

[60] Loudon K Treweek S Sullivan F Donnan P Thorpe KE Zwarenstein M. The Precis-2 tool: designing trials that are fit for purpose. BMJ. (2015) 350:h2147. 10.1136/bmj.h2147 25956159

[61] Zhang YQ Jiao RM Witt CM Lao L Liu J-P Thabane L . How to design high quality acupuncture trials—a consensus informed by evidence. BMJ. (2022) 376:e067476. 10.1136/bmj-2021-067476 35354583 PMC8965655

