# Supplementary material for: Acupuncture for neuropathic pain: A meta-analysis of randomized control trials
Source: Front Neurol. 2023 Jan 9;13:1076993. doi: 10.3389/fneur.2022.1076993 (PMC9868276; doi:10.3389/fneur.2022.1076993)
Supplement: Supplementary file 1 [file Data_Sheet_1.DOCX]

| **Section and Topic** | **Item #** | **Checklist item** | **Location where item is reported** |
| --- | --- | --- | --- |
| **TITLE** | | |  |
| Title | 1 | Acupuncture for Neuropathic Pain: A Meta-Analysis of Randomized Control Trials | 1 |
| **ABSTRACT** | | |  |
| Abstract | 2 | See the PRISMA 2020 for Abstracts checklist. | / |
| **INTRODUCTION** | | |  |
| Rationale | 3 | The International Association for the Study of Pain (IASP) recently updated the definition of neuropathic pain (NP) as "pain caused by a lesion or disease of the somatosensory nervous system"(1). NP has both the “positive” symptoms (hyperalgesia, allodynia, shooting pain, burning pain, especially at rest) that require therapy and the “negative” symptoms (sensory deficits like hypalgesia and hypaesthesia) that cannot be treated with medication(2). A survey of the general population sampled by multimodal recruitment in 2017 reported that about 10% people in the United States suffer from NP(3). With the ageing of the global population, NP is extremely likely to increase(4). Moreover, NP seriously affects patients’ quality of life(5). Specifically, patients with NP have anxiety, depression, poor sleep, psychological disorder, physical disability, and social dysfunction(6-10). An observational study on the economic burden of patients with NP at all pain intensity levels in the United States showed that the annualized direct medical expenses to payers were $6,016, the annualized direct expenses to subjects were $2,219, and the annualized indirect expenses of each subject were $19,000(11).  To date, the medications of NP is focus on five categories including serotonin/norepinephrine-modulating antidepressants, Na-blocker anticonvulsants, Ca-modulator anti-convulsants, tramadol, and opioids, and two types of topical medicine including local anesthetics and capsaicin(12). However, pharmacological treatment is not very effective for NP for the patients keep reporting inadequate pain relief, and a progressive decrease in the estimated effect of NP drugs has been reported(13). In addition, a randomized controlled trial (RCT) on the safety of antiepileptics and antidepressants for NP showed that the incidence of any treatment emergent adverse event (AE) ranged from 7% to 91.7% compared with the placebo groups, and the dizziness, drowsiness, nausea, and constipation were the most commonly reported AEs(14). Despite the lack of evidence to show beneficial effects, clinical trials on novel analgesic medicine to treat NP are lacking in recent years(15, 16).  Given the situation that NP is mostly chronic, which means that long-term management is required, therefore, it is critical to develop treatment protocol concentrating on improving efficacy and safety monitoring(17). As an alternative and complementary medicine, acupuncture refers to inserting needles on acupoints or specific parts of the human body at different depths by various manipulations(18). Widespread and effective application of acupuncture were encouraged in the clinical treatment of NP in recent years(19-27). In addition, a meta-analysis reported that acupuncture could be considered the safer therapy in medications, with the reasons that serious AEs were rare, and the most common AEs were mild(28). | 2 |
| Objectives | 4 | However, some researchers consider the difference between acupuncture and sham acupuncture as not being clinically significant(29). A systematic review and meta-analysis(30) on acupuncture in the treatment of NP in adults was published in 2017 suggested that it is challenging to either support or refute the effect of acupuncture for NP due to limited data available. Considering the widespread and effective application of acupuncture in the clinical treatment of NP, the previous meta-analysis conclusions need to be further verified. Therefore, this study was conducted to explore the effect on pain intensity and safety of acupuncture in patients with NP. | 2-3 |
| **METHODS** | | |  |
| Eligibility criteria | 5 | Studies were eligible if met the following criteria: 1) RCTs evaluating acupuncture for NP; 2) participants with the diagnosis of NP; 3) acupuncture treatments as the main observational therapies (including traditional acupuncture, auricular acupuncture, ear needle, abdominal acupuncture, etc); 3) the control group could be conventional treatment, sham acupuncture, or blank control; 4) pain change variables including but not limited to visual analog scale (VAS) score, Numeric Rating Scale (NRS) , Brief Pain Inventory-Short Form (BPI-SF) worst pain score. Limited to reports in English language, AEs was used to assess the safety of acupuncture therapies. The following studies were excluded: 1) conference abstracts, case report, protocol, reviews, animal or cellular level experiments; 2) duplicated literature; 3) studies with insufficient data; 4) trials with acupuncture therapy in control group; 5) studies using methods not based on TCM theory like dry needle; 6) articles on moxibustion, cupping, herbal medicine, laser acupuncture, and any combination of the above; 7) literature not published in English language. | 3 |
| Information sources | 6 | An encompassing search of specific authoritative databases inception to May 2022, in English, was conducted. The databases were as follow: Scopus, Ovid EMBASE, Ovid Cochrane Database of Systematic Reviews, Ovid Cochrane Central Register of Controlled Trials, Ovid MEDLINE(R) and Epub Ahead of Print and In-Process & Other Non-Indexed Citations, and Daily. The search strategy was designed and performed by a professional librarian. The procedure was described in the supplementary information of this article. | 3 |
| Search strategy | 7 | The procedure was described in the supplementary information of this article. | 3 |
| Selection process | 8 | Full text articles were retrieved after screening based on titles and abstracts of all articles according to the criteria by two independent researchers. In addition, discussion was carried out in case of disagreements, and a third party helped to reach a consensus if necessary. | 3 |
| Data collection process | 9 | Data were collected independently by two researchers using Excel tables from every included study and reviewed by a third party. | 3 |
| Data items | 10a | Pain change variables including but not limited to visual analog scale (VAS) score, Numeric Rating Scale (NRS) , Brief Pain Inventory-Short Form (BPI-SF) worst pain score. | 3 |
|  | 10b | 1) RCTs evaluating acupuncture for NP; 2) participants with the diagnosis of NP; 3) acupuncture treatments as the main observational therapies (including traditional acupuncture, auricular acupuncture, ear needle, abdominal acupuncture, etc); 3) the control group could be conventional treatment, sham acupuncture, or blank control. | 3 |
| Study risk of bias assessment | 11 | Studies were evaluated with the Cochrane risk of bias assessment tool(31) by the two researchers independently. Related evaluations were as follow: sequence generation, allocation concealment, blinding, incomplete outcome data, selective outcome reporting, and other sources. Moreover, each part of evaluation was defined as low, high, or unclear risk of bias. A third party help reach a consensus if there was a conflict. | 4 |
| Effect measures | 12 | The mean difference (MD) before and after treatment was used to pool differences between the experimental and control groups for each study. | 3 |
| Synthesis methods | 13a | Data were collected independently by two researchers using Excel tables from every included study and reviewed by a third party. The collected information contained the first author’s name, year of publication, subjects. age, condition, sample size, interventions, sessions, outcome measures, follow-up, the selected acupoints of treatment, etc. The pain intensity outcomes were recorded as continuous variables. | 3 |
|  | 13b | Outcome data were expressed with the standardized mean difference (SMD) and 95% confidence interval (CI) to standardize the study results into a uniform scale. | 3 |
|  | 13c | Data were collected independently by two researchers using Excel tables from every included study and reviewed by a third party. And turned detailed information into tables or figures. | 3 |
|  | 13d | All the analyses were carried out by RevMan 5.3. Qualitative analysis was carried out if extraction was insufficient to conduct meta-analysis. When analyzing continuous variables, mean difference with 95% CIs were calculated with heterogeneity tested by *I^2^* test. Date was combined by fixed effect model when *I^2^*<50%. Otherwise, random effect model was carried out. There was a significant difference if *P*<0.05 between two groups. | 4 |
|  | 13e | Subgroup analysis or sensitivity analysis could help find out the sources of heterogeneity. Furthermore, a descriptive analysis was conducted when the reasons of heterogeneity could not be determined. | 4 |
|  | 13f | Funnel plot was applied to assess publication bias. | 4 |
| Reporting bias assessment | 14 | Studies were evaluated with the Cochrane risk of bias assessment tool^25^ by the two researchers independently. Related evaluations were as follow: sequence generation, allocation concealment, blinding, incomplete outcome data, selective outcome reporting, and other sources. Moreover, each part of evaluation was defined as low, high, or unclear risk of bias. A third party help reach a consensus if there was a conflict. | 4 |
| Certainty assessment | 15 | We rated the general quality of outcome with the classification of GRADEpro GDT (<https://www.gradepro.org/>) in the following areas: study design, risk of bias, inconsistency, indirectness, imprecision, and other considerations. | 4 |
| **RESULTS** | | |  |
| Study selection | 16a | We yielded 5813 studies after searching the databases. In addition to three duplicates, 5134 records were removed for irrelevant results after screening. A total of 46 full-text manuscripts were further screened. After excluding 30 reports that did not meet the inclusion criteria, 16 studies with 1021 patients with NP in English were included in the systematic review. Finally, eight trials with 338 participants were conducted with a meta-analysis. The selection flow of trials is presented in Figure 1. | 4 |
|  | 16b | We yielded 5813 studies after searching the databases. In addition to three duplicates, 5134 records were removed for irrelevant results after screening. A total of 46 full-text manuscripts were further screened. After excluding 30 reports that did not meet the inclusion criteria, 16 studies with 1021 patients with NP in English were included in the systematic review. Finally, eight trials with 338 participants were conducted with a meta-analysis. The selection flow of trials is presented in Figure 1. | 4 |
| Study characteristics | 17 | All the included studies were RCTs, and two(24, 26) of which were multicenter RCTs, including one study(24) located in four centers in South Korea, the other study(26) located in Iran and China. Besides, there were two(20, 21) from Taiwan, China, five(23, 27, 32-34) from the United States, three(19, 25, 35) from Iran, two(22, 36) from the United Kingdom, and one from Croatia(37) and Italy(38), individually. The included studies contained various types of NP including one article on each of the following: post-herpetic neuralgia(36), chronic sciatic pain(19), idiopathic neuropathy(38), burning mouth syndrome(37), spinal cord injury(23), migraine(35) respectively; in addition, there were three studies on carpal tunnel syndrome(20, 21, 25) or diabetic painful neuropathy(22, 24, 38) and five(26, 27, 32-34) on chemotherapy-induced peripheral neuropathy. Conventional acupuncture was used in eight trials(20-22, 25-27, 36, 37), EA was used in four trials(19, 24, 32, 38), and auricular acupuncture was used in five trials(23, 33-36). The sessions of the interventions varied from four to 12 weeks, and durations of treatments were from 20 to 30 minutes except semi-permanent auricular acupuncture. A total of 10 studies(21, 23-27, 32-34, 38) reported follow-up investigations, nine(23-27, 32, 33, 38) of which ranged from four weeks to 12 weeks, and one(21) of which was one year. Seven studies(20, 22, 25, 26, 32, 34, 35) mentioned the background of acupuncture practitioners, of which six(20, 22, 25, 32, 34, 35) were carried out by acupuncturists and one(26) by physicians with acupuncture experience. Moreover, 10 studies(19-27) reported positive effects, five studies(32, 34, 36-38) reported negative effects, and one study(33) did not report any clear effect on pain intensity. Detailed information is presented in Table 1. | 5-6 |
| Risk of bias in studies | 18 | Twelve(19-22, 24-26, 33, 36, 37) of the included RCTs were evaluated with a low risk of bias of randomization sequence generation with detailed description of randomization methods. Seven RCTs(20-22, 24, 33, 34, 36) used computer-generated randomization list, one trial(37) used a simple randomization method of flipping a coin, one trial(25) used the random numbers table and three trials(25, 27, 35) used a random allocation software. Four trials(19, 23, 32, 38) lacked detailed information resulted in an unclear risk of bias of randomization. Three trials(22, 24, 26) placing their information sequentially with sealed opaque envelopes, were evaluated with the low risk of bias of allocation concealment. One trial(25) used an open randomization of random numbers table which resulted in a high risk of bias of concealment. The remaining 12 trials(19-21, 23, 27, 32-38) without sufficient description in detail were regarded as an unclear risk of bias of allocation concealment. None of the trials were double-blinded because the acupuncturists were not blinded. Nine trials(19, 21, 24, 26, 33-36, 38) was single-blinded in outcome assessment, five trials(20, 22, 25, 27, 37) did not describe methods of blinding, and two trials(23, 32) indicated that assessors were not blinded. Three trials(25, 26, 35) reported no attrition in follow-up studies, while seven trials(21-24, 27, 32, 34) were intention-to-treat (ITT) analysis. However, one trial(33) did not address the loss of follow-up treatments, while five trials(19, 20, 36-38) had no description of the loss of follow-up. Nine trials(20, 22, 25-27, 32-34) registered online previously with certain outcomes led to low risk of bias in selective reporting. One trial(24) did not report the complete outcome data. Therefore, we were unable to extract the data for meta-analysis, resulting in unclear risk of bias. The left trials(19, 21, 23, 36-38) had an unclear risk of bias without detailed report. In other sources of bias, all trials were evaluated with a low risk. The brief information was shown in Figure 2&3. | 10 |
| Results of individual studies | 19 | Information was shown in Figure 4. | 12 |
| Results of syntheses | 20a | High risk of bias due to lack of allocation concealment, blinding and incomplete outcome data, moderate heterogeneity and outcome with wide CI. | 15 |
|  | 20b | Changes in pain intensity (including changes of VAS, NRS and BPI-SF worst pain score) occurred in eight RCTs(22, 23, 26, 32, 33, 35, 37, 38) with 338 participants. They investigated the effect of acupuncture on changes in pain intensity, including four trials(22, 26, 37, 38) on conventional manual acupuncture, three trials(23, 33, 35) on auricular acupuncture and one trial(32) on EA. Using a random effect model among the results (*P=0.02*, *I^2^* =59%), a significant effect was shown in changes in pain intensity in the acupuncture group (SMD -0.59, 95% CI -0.95 to -0.23, *P*=0.001) (Figure 4). Eight trials(19-21, 24, 27, 33, 34, 36) were not pooled in the meta-analysis because one trial(24) did not report complete data and seven trials(19-21, 27, 33, 34, 36) did not report relative outcome.  Subgroup analysis was used to verified if different interventions of control group would affect the changes in pain intensity. Five trials(22, 32, 33, 35, 38) including 238 patients using a random effect model indicated that acupuncture was more effective in improving changes in pain intensity than sham acupuncture (SMD -0.54, 95% CI -0.95 to -0.13, *P*=0.01). Two trials(26, 37) evaluated the effect on changes in pain intensity with a random effect model among 80 patients in the comparison of acupuncture and conventional treatments including clonazepam in study of Jurisic Kvesic 2015 et al(37) and vit B1 and gabapentin in study of Iravani 2020 et al(26), no significant difference existed (SMD -0.61, 95% CI -1.83 to 0.61, *P*=0.33). One trial(23) compared acupuncture with blank control evaluating the effect on changes in pain intensity with a significant difference (Figure 5).  According to the subgroup analysis, the acupuncture group seemed worse than conventional treatments, but there were benefits when compared to sham intervention. or blank control in treating NP. | 12 |
|  | 20c | The sensitivity analysis showed that studies of Iravani et al(26) and Habibabadi et al(35) may be the main cause of heterogeneity, because *I^2^* went down to 0% after removing them. Different types of NP, acupoints and sessions may be the reasons of heterogeneity. | 13 |
|  | 20d | The sensitivity analysis showed that studies of Iravani et al(26) and Habibabadi et al(35) may be the main cause of heterogeneity, because *I^2^* went down to 0% after removing them. Different types of NP, acupoints and sessions may be the reasons of heterogeneity. Funnel plot of changes in pain intensity was symmetric, which means no publication bias was detected (Figure 6). | 13 |
| Reporting biases | 21 | However, one trial(33) did not address the loss of follow-up treatments, while five trials(19, 20, 36-38) had no description of the loss of follow-up. Nine trials(20, 22, 25-27, 32-34) registered online previously with certain outcomes led to low risk of bias in selective reporting. One trial(24) did not report the complete outcome data. Therefore, we were unable to extract the data for meta-analysis, resulting in unclear risk of bias. The left trials(19, 21, 23, 36-38) had an unclear risk of bias without detailed report. | 10 |
| Certainty of evidence | 22 | The GRADE was rated as "very low". | 15 |
| **DISCUSSION** | | |  |
| Discussion | 23a | This study included a systematic review of 16 RCTs with 1021 patients and a meta-analysis of eight studies with 338 subjects assessing the effect on pain intensity and safety of acupuncture in patients with NP. Our findings from the qualitative analysis of systematic review showed an unclear effect of acupuncture on improving pain intensity in patients with NP, because 10 studies(19-27) reported positive effects, five studies(32, 34, 36-38) reported negative effects, and one study(33) did not report any clear effect on pain intensity. However, results of the meta-analysis indicated that acupuncture was an effective intervention for patients with NP. Additionally, 11 trials(20-22, 24, 26, 27, 32-34, 37, 38) reported safety conditions, and acupuncture induced AEs which were mentioned above were mild and reversible, indicating that acupuncture is a relatively safe intervention for patients with NP.  According to the results of the subgroup analysis, acupuncture had higher effectiveness than sham acupuncture or blank control, which means that acupuncture is also an effective treatment for NP. Our study also showed that conventional manual acupuncture, EA, auricular acupuncture all contributed to pain relief. However, there was no significant difference between acupuncture and conventional treatments for NP. Moreover, it is notable that compared with the side effects of conventional treatments, acupuncture induced AEs were mild and reversible. Therefore, acupuncture may be beneficial to improve pain intensity of patients with NP in a relatively safe means, and as a complementary part to provide more specific evidence to improve clinical practice. The results of the sensitivity analysis and funnel plot showed that the effect of acupuncture on the changes in pain intensity in patients with NP was robust. Furthermore, the “very low” GRADE results of changes in pain intensity may suggest this treatment to clinical practice with a recommendation level of “very low”. Ultimately, the interpretation of our results should be performed cautiously due to low methodological quality.  Developing after nerve injuries, NP occurs in deleterious changes in damaged neurons, and goes along the nociceptive and descending modulatory pathways of the central nervous system(39). Sensitization of nociceptive pathways mainly raises by maladaptive alterations of structural, cells interactions and molecular signaling, including changes in activation of immune cells, glial-derived mediators, ion channels and epigenetic regulation(40). A trial found that EA in low frequency ameliorated NP by stimulating the spinal microglial expression of IL-10 and subsequent β-endorphin with NP rats caused by tightly ligating the L5/6 spinal nerves(41). In addition, a trial suggested that EA improved tactile allodynia after peripheral nerve injury by inhibiting excessive expression of IFN-γ in the spinal cord and subsequent P2X4R with a chronic constriction injury (CCI) rat model for NP (42). Moreover, a study found that EA can modulate miR-214 to inhibit neuronal apoptosis by targeting Bax and prevented the expression of Nav1.3 channel in rats of SCI(43). Furthelrmore, a study suggested that acupuncture can alleviated chronic pain-induced comorbid conditions by changing DNA methylation of Nr4a1, Rasgrp1, Rassf1, and Chkb in the PFC with NP rats caused by left partial sciatic nerve ligation(44). Currently, the mechanism of acupuncture for NP has not met an agreement, thus more concentration is needed to focus on how acupuncture relieve NP. | 16-17 |
|  | 23b | However, there were also some limitations of this study. Firstly, in the qualitative analysis of systematic review, six(32-34, 36-38) of the 16 trials indicated a negative or ambiguous effect on pain intensity of acupuncture for NP. Secondly, the outcomes of life quality evaluation were inadequate to pooled to perform the meta-analysis. Thirdly, GRADE was rated as “very low”. And the quality of total studies was low, especially in the area of allocation concealment and participants and personnel blindness. | 17 |
|  | 23c | However, there were also some limitations of this study. Firstly, in the qualitative analysis of systematic review, six(32-34, 36-38) of the 16 trials indicated a negative or ambiguous effect on pain intensity of acupuncture for NP. Secondly, the outcomes of life quality evaluation were inadequate to pooled to perform the meta-analysis. Thirdly, GRADE was rated as “very low”. And the quality of total studies was low, especially in the area of allocation concealment and participants and personnel blindness. | 17 |
|  | 23d | Our study has a multiple of strengths. Firstly, our review focused on the effect of using acupuncture alone, so we excluded the studies of mixed therapies and conducted a subgroup study of sham acupuncture or blank control in the control group to verify whether acupuncture is effective for NP. Secondly, a previously published meta-analysis(30) showed it is challenging to either support or refute the effect of acupuncture for NP, nevertheless, the study only included two manual acupuncture RCTs(22, 45) on pain intensity in its meta-analysis. In contrast, we include more RCTs with a larger sample size and more acupuncture manipulations. Moreover, compared with the previous study(30), both CNP and PNP patients were included in our study, which may more strongly support the hypothesis that acupuncture on NP is effective. Thirdly, many studies performing at multiple locations and in different countries, covering a more ethnically and culturally diverse sample, which may reduce selection bias and improve external validity. Fourthly, sensitivity analysis and funnel plot were conducted, demonstrating that the meta-analysis was stable and irreversible without publication bias. Fifthly, most of studies were longitudinal, and one of them was followed for one year. To some extent, our study provided supporting evidence for the clinical practice of acupuncture in the treatment of patients with NP.  More high-quality studies on acupuncture for patients with NP are needed to enlarge the sample size and reduce bias. Longer follow-up trials are required to observe the long-term effect of acupuncture in the treatment of NP. Consolidated Standards of Reporting Trials (CONSORT) statement and STRICTA checklists(46, 47) should be followed in future studies. In order to achieve double-blinding, standardized trial design, timely data storage system and well-coordinated team are needed to help performed sham intervention successfully, which can refer to pragmatic-explanatory continuum indicator summary (PRECIS) or PRECIS-2(48-50). | 17,18 |
| **OTHER INFORMATION** | | |  |
| Registration and protocol | 24a | The protocol has been registered in the PROSPERO database with registration number: CRD42022306461. | 3 |
|  | 24b | The protocol has been registered in the PROSPERO database. | 3 |
|  | 24c | Our one of four comparisons of selection criteria set in protocol included acupuncture combined with other therapies compared with other active therapies. But we decided to amend the comparison as follow: acupuncture alone in treatment group compared with the control group which could be conventional treatment, sham acupuncture, or blank control in final manuscript. Because we considered that the comparison of combination of acupuncture and other active therapies would cover up the real effect of acupuncture, which may reduce the credibility of our conclusion. Thus, our subgroup analysis were correspondingly amended as:1)acupuncture vs. sham acupuncture; 2) acupuncture vs. blank control; 3) acupuncture vs. conventional treatment. | / |
| Support | 25 | This study is supported by Chinese Medicine Key Medical Specialties Construction Project of Shenzhen Municipal Health Commission (Grant NO. ZYTS019). | 18 |
| Competing interests | 26 | The authors declare that there are no conflicts of interest in this work. | 18 |
| Availability of data, code and other materials | 27 | The procedure of search strategy was described in the supplementary information of this article. | 3 |

*From:*  Page MJ, McKenzie JE, Bossuyt PM, Boutron I, Hoffmann TC, Mulrow CD, et al. The PRISMA 2020 statement: an updated guideline for reporting systematic reviews. BMJ 2021;372:n71. doi: 10.1136/bmj.n71

For more information, visit: <http://www.prisma-statement.org/>

**PRISMA 2020 for Abstracts Checklist**


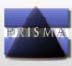


| **Section and Topic** | **Item #** | **Checklist item** | **Reported (Yes/No)** |
| --- | --- | --- | --- |
| **TITLE** | | |  |
| Title | 1 | Acupuncture for Neuropathic Pain: A Meta-Analysis of Randomized Control Trials | Yes |
| **BACKGROUND** | | |  |
| Objectives | 2 | Neuropathic pain (NP) is expected to increase due to the high risk of global population aging. Acupuncture has definite clinical effect on NP. Therefore, a systematic review and meta-analysis was conducted to evaluate the effect on pain intensity and safety of acupuncture in patients with NP. | Yes |
| **METHODS** | | |  |
| Eligibility criteria | 3 | All the randomized controlled trials regarding acupuncture treatment of NP will be included. | Yes |
| Information sources | 4 | An encompassing search of specific authoritative databases in English, from their inception to May 2022, was performed. The databases were as follow: Scopus, Ovid EMBASE, Ovid Cochrane Database of Systematic Reviews, Ovid Cochrane Central Register of Controlled Trials, Ovid MEDLINE(R) and Epub Ahead of Print and In-Process & Other Non-Indexed Citations, and Daily. | Yes |
| Risk of bias | 5 | Methodological quality assessment of the included trials was assessed based on the risk of bias from Cochrane handbook. | Yes |
| Synthesis of results | 6 | A meta-analysis was performed for changes in pain intensity. In addition, sensitivity analysis, subgroup analysis and funnel plot were also carried out. | Yes |
| **RESULTS** | | |  |
| Included studies | 7 | A total of 16 studies with 1021 patients with NP were evaluated in systematic review. Eleven studies mentioned the safety conditions, of which induced by acupuncture were all mild and reversible. | Yes |
| Synthesis of results | 8 | A total of 16 studies with 1021 patients with NP were evaluated in systematic review. According to results of over-all meta-analysis in eight RCTs with 338 participants, the acupuncture group was better than the control group in improving changes in pain intensity (SMD -0.59, 95% CI -0.95 to -0.23, *P*=0.001). In subgroups analysis, five trials indicated that acupuncture was more effective in improving changes in pain intensity than sham acupuncture (SMD -0.54, 95% CI -0.95 to -0.13, P=0.01), two trials evaluated the effect on changes in pain intensity in the comparison of acupuncture and conventional treatments, no significant difference existed (SMD -0.61, 95% CI -1.83 to 0.61, P=0.33), and one trial compared acupuncture with blank control evaluating the effect on changes in pain intensity with a significant difference. Eleven studies mentioned the safety conditions, of which induced by acupuncture which were mentioned above were mild and reversible. Both the sensitivity analysis and funnel plot analysis showed that the meta-analysis was stable and irreversible without publication bias. The GRADE was rated as "very low". | Yes |
| **DISCUSSION** | | |  |
| Limitations of evidence | 9 | Both the sensitivity analysis and funnel plot analysis showed that the meta-analysis was stable and irreversible without publication bias. The GRADE was rated as "very low". | Yes |
| Interpretation | 10 | The acupuncture group seemed worse than conventional treatments on changes in pain intensity, but there were benefits when compared to sham intervention or blank control in treating NP. The acupuncture induced adverse events were mild and reversible. However, the interpretation of our results should be performed cautiously due to low methodological quality. | Yes |
| **OTHER** | | |  |
| Funding | 11 | This study is supported by Chinese Medicine Key Medical Specialties Construction Project of Shenzhen Municipal Health Commission (Grant NO. ZYTS019). | Yes |
| Registration | 12 | The protocol has been registered in the PROSPERO database with registration number: CRD4202230646. | Yes |

*From:*  Page MJ, McKenzie JE, Bossuyt PM, Boutron I, Hoffmann TC, Mulrow CD, et al. The PRISMA 2020 statement: an updated guideline for reporting systematic reviews. BMJ 2021;372:n71. doi: 10.1136/bmj.n71

For more information, visit: <http://www.prisma-statement.org/>
