# Supplementary material for: Acupuncture for neuropathic pain: A meta-analysis of randomized control trials
Source: Front Neurol. 2023 Jan 9;13:1076993. doi: 10.3389/fneur.2022.1076993 (PMC9868276; doi:10.3389/fneur.2022.1076993)
Supplement: Supplementary file 2 [file Data_Sheet_2.DOCX]

Ovid

Database(s): EBM Reviews - Cochrane Central Register of Controlled Trials April 2022, EBM Reviews - Cochrane Database of Systematic Reviews 2005 to May 5, 2022, Embase 1974 to 2022 May 04, Ovid MEDLINE(R) and Epub Ahead of Print, In-Process, In-Data-Review & Other Non-Indexed Citations, Daily and Versions 1946 to May 04, 2022
Search Strategy:

| **#** | **Searches** | **Results** |
| --- | --- | --- |
| 1 | Acupuncture/ or exp Acupuncture Therapy/ | 86140 |
| 2 | (acupoint* or acupressure or acupuncture or auriculotherap* or "ear needl*" or electroacupuncture or "electro-acupuncture" or "elongated needl*" or "fire needl*" or meridian* or moxibustion or needling or Shiatsu or "Tui Na" or "warm needl*").ti,ab,hw,kw. | 129451 |
| 3 | 1 or 2 | 129470 |
| 4 | exp neuropathic pain/ | 62939 |
| 5 | exp Neuralgia/ | 142363 |
| 6 | exp Peripheral Nervous System Diseases/ | 244739 |
| 7 | exp Somatosensory Disorders/ | 137141 |
| 8 | exp Herpes Zoster/ | 41114 |
| 9 | exp Causalgia/ | 1130 |
| 10 | exp Trigeminal Neuralgia/ | 18947 |
| 11 | exp Phantom Limb/ | 4958 |
| 12 | exp Spinal Cord Injuries/ | 139249 |
| 13 | exp Paraplegia/ | 37967 |
| 14 | exp Quadriplegia/ | 27609 |
| 15 | exp Carpal Tunnel Syndrome/ | 26654 |
| 16 | exp Bell Palsy/ | 5660 |
| 17 | (((neur* or nerv*) adj5 (compress* or damag*)) or ((pain* or discomfort*) adj5 (central or complex or rheumat* or muscl* or muscul* or myofasci* or nerv* or neuralg* or neuropath*)) or "bell palsies" or "bell palsy" or "bells palsies" or "bells palsy" or "brown presentation" or "brown sequard disease" or "Brown Sequard syndrome*" or "Brown-Sequards syndrome*" or "carpal tunnel syndrome*" or causalgia or "central cord syndrome*" or "central spinal cord syndrome*" or "complex regional pain syndrome type ii" or "crps type ii" or "deafferentation pain" or "fothergill disease" or "herpes zoster" or "herpetic facial paralyses" or "herpetic facial paralysis" or "idiopathic acute facial neuropathy" or "idiopathic facial paralyses" or "idiopathic facial paralysis" or "medullary transverse lesion*" or mononeuropath* or neuralgia* or neurodynia* or "neuropathic pain*" or neuropathy or Paraplegia* or "phantom limb*" or polyneuropath* or "Post Traumatic Myelopath*" or Quadriplegia* or shingles or "Somatosensory Disorder*" or "Spinal Cord Contusion*" or "Spinal Cord Injur*" or "Spinal Cord Laceration*" or "Spinal Cord Transection*" or "spinal cord transsection*" or "spinal cord transverse lesion*" or "Spinal Cord Trauma*" or "tic doloureux" or "tic douloureux" or "transverse cord lesion*" or "transverse lesion*" or "transverse spinal cord lesion*" or "Traumatic Myelopath*" or "type ii complex regional pain syndrome*").ti,ab,hw,kw. | 1003563 |
| 18 | or/4-17 | 1213032 |
| 19 | 3 and 18 | 12802 |
| 20 | exp evidence based medicine/ | 1583885 |
| 21 | exp meta analysis/ | 405847 |
| 22 | exp Meta-Analysis as Topic/ | 74459 |
| 23 | exp "systematic review"/ | 539040 |
| 24 | exp Guideline/ or exp Practice Guideline/ | 678633 |
| 25 | exp controlled study/ | 8973622 |
| 26 | exp Randomized Controlled Trial/ | 1278270 |
| 27 | "Randomised controlled trials".sd. | 240188 |
| 28 | exp triple blind procedure/ | 328 |
| 29 | exp Double-Blind Method/ | 513665 |
| 30 | exp Single-Blind Method/ | 100987 |
| 31 | exp latin square design/ | 400 |
| 32 | exp Placebos/ | 443649 |
| 33 | exp Placebo Effect/ | 13869 |
| 34 | exp comparative study/ | 3464226 |
| 35 | exp intervention studies/ | 55092 |
| 36 | exp Cross-Sectional Studies/ | 910297 |
| 37 | exp Cross-Over Studies/ | 164182 |
| 38 | exp Cohort Studies/ | 3344904 |
| 39 | exp longitudinal study/ | 336691 |
| 40 | exp retrospective study/ | 2276292 |
| 41 | exp prospective study/ | 1490319 |
| 42 | exp clinical trial/ | 2634288 |
| 43 | clinical study/ | 162841 |
| 44 | exp case-control studies/ | 1540574 |
| 45 | exp confidence interval/ | 176959 |
| 46 | exp multivariate analysis/ | 605828 |
| 47 | ((evidence adj based) or (meta adj analys*) or (systematic* adj3 review*) or guideline* or (control* adj3 study) or (control* adj3 trial) or (randomized adj3 study) or (randomized adj3 trial) or (randomised adj3 study) or (randomised adj3 trial) or "pragmatic clinical trial" or (doubl* adj blind*) or (doubl* adj mask*) or (singl* adj blind*) or (singl* adj mask*) or (tripl* adj blind*) or (tripl* adj mask*) or (trebl* adj blind*) or (trebl* adj mask*) or "latin square" or placebo* or nocebo* or multivariate or "comparative study" or "comparative survey" or "comparative analysis" or (intervention* adj2 study) or (intervention* adj2 trial) or "cross-sectional study" or "cross-sectional analysis" or "cross-sectional survey" or "cross-sectional design" or "prevalence study" or "prevalence analysis" or "prevalence survey" or "disease frequency study" or "disease frequency analysis" or "disease frequency survey" or crossover or "cross-over" or cohort* or "longitudinal study" or "longitudinal survey" or "longitudinal analysis" or "longitudinal evaluation" or longitudinal* or ((retrospective or "ex post facto") adj3 (study or survey or analysis or design)) or retrospectiv* or "prospective study" or "prospective survey" or "prospective analysis" or prospectiv* or "concurrent study" or "concurrent survey" or "concurrent analysis" or "clinical study" or "clinical trial" or "case control study" or "case base study" or "case referrent study" or "case referent study" or "case referent study" or "case compeer study" or "case comparison study" or "matched case control" or "multicenter study" or "multi-center study" or "odds ratio" or "confidence interval" or "change analysis" or ((study or trial or random* or control*) and compar*)).mp,pt. | 27528229 |
| 48 | or/20-47 | 28269785 |
| 49 | 19 and 48 | 8319 |
| 50 | (exp animals/ or exp nonhuman/) not exp humans/ | 11809080 |
| 51 | ((alpaca or alpacas or amphibian or amphibians or animal or animals or antelope or armadillo or armadillos or avian or baboon or baboons or beagle or beagles or bee or bees or bird or birds or bison or bovine or buffalo or buffaloes or buffalos or "c elegans" or "Caenorhabditis elegans" or camel or camels or canine or canines or carp or cats or cattle or chick or chicken or chickens or chicks or chimp or chimpanze or chimpanzees or chimps or cow or cows or "D melanogaster" or "dairy calf" or "dairy calves" or deer or dog or dogs or donkey or donkeys or drosophila or "Drosophila melanogaster" or duck or duckling or ducklings or ducks or equid or equids or equine or equines or feline or felines or ferret or ferrets or finch or finches or fish or flatworm or flatworms or fox or foxes or frog or frogs or "fruit flies" or "fruit fly" or "G mellonella" or "Galleria mellonella" or geese or gerbil or gerbils or goat or goats or goose or gorilla or gorillas or hamster or hamsters or hare or hares or heifer or heifers or horse or horses or insect or insects or jellyfish or kangaroo or kangaroos or kitten or kittens or lagomorph or lagomorphs or lamb or lambs or llama or llamas or macaque or macaques or macaw or macaws or marmoset or marmosets or mice or minipig or minipigs or mink or minks or monkey or monkeys or mouse or mule or mules or nematode or nematodes or octopus or octopuses or orangutan or "orang-utan" or orangutans or "orang-utans" or oxen or parrot or parrots or pig or pigeon or pigeons or piglet or piglets or pigs or porcine or primate or primates or quail or rabbit or rabbits or rat or rats or reptile or reptiles or rodent or rodents or ruminant or ruminants or salmon or sheep or shrimp or slug or slugs or swine or tamarin or tamarins or toad or toads or trout or urchin or urchins or vole or voles or waxworm or waxworms or worm or worms or xenopus or "zebra fish" or zebrafish) not (human or humans or patient or patients)).ti,ab,hw,kw. | 10085714 |
| 52 | 49 not (50 or 51) | 7432 |
| 53 | (case adj3 report).mp,pt. | 3364427 |
| 54 | 52 not 53 | 7234 |
| 55 | limit 54 to (editorial or erratum or note or addresses or autobiography or bibliography or biography or blogs or comment or dictionary or directory or interactive tutorial or interview or lectures or legal cases or legislation or news or newspaper article or overall or patient education handout or periodical index or portraits or published erratum or video-audio media or webcasts) [Limit not valid in CCTR,CDSR,Embase,Ovid MEDLINE(R),Ovid MEDLINE(R) Daily Update,Ovid MEDLINE(R) PubMed not MEDLINE,Ovid MEDLINE(R) In-Process,Ovid MEDLINE(R) Publisher; records were retained] | 166 |
| 56 | from 55 keep 2-20 | 19 |
| 57 | (54 not 55) or 56 | 7087 |
| 58 | limit 57 to yr="2015 -Current" | 3643 |
| 59 | remove duplicates from 58 | 2501 |
| 60 | 57 not 58 | 3444 |
| 61 | remove duplicates from 60 | 2422 |
| 62 | 59 or 61 | 4923 |

Scopus

1 TITLE-ABS-KEY(acupoint* OR acupressure OR acupuncture OR auriculotherap* OR "ear needl*" OR electroacupuncture OR "electro-acupuncture" OR "elongated needl*" OR "fire needl*" OR meridian* OR moxibustion OR needling OR Shiatsu OR "Tui Na" OR "warm needl*")

2 TITLE-ABS-KEY(((neur* or nerv*) W/5 (compress* or damag*)) OR ((pain* or discomfort*) W/5 (central or complex or rheumat* or muscl* or muscul* or myofasci* or nerv* or neuralg* or neuropath*)) OR "bell palsies" OR "bell palsy" OR "bells palsies" OR "bells palsy" OR "brown presentation" OR "brown sequard disease" OR "Brown Sequard syndrome*" OR "Brown-Sequards syndrome*" OR "carpal tunnel syndrome*" OR causalgia OR "central cord syndrome*" OR "central spinal cord syndrome*" OR "complex regional pain syndrome type ii" OR "crps type ii" OR "deafferentation pain" OR "fothergill disease" OR "herpes zoster" OR "herpetic facial paralyses" OR "herpetic facial paralysis" OR "idiopathic acute facial neuropathy" OR "idiopathic facial paralyses" OR "idiopathic facial paralysis" OR "medullary transverse lesion*" OR mononeuropath* OR neuralgia* OR neurodynia* OR "neuropathic pain*" OR neuropathy OR Paraplegia* OR "phantom limb*" OR polyneuropath* OR "Post Traumatic Myelopath*" OR Quadriplegia* OR shingles OR "Somatosensory Disorder*" OR "Spinal Cord Contusion*" OR "Spinal Cord Injur*" OR "Spinal Cord Laceration*" OR "Spinal Cord Transection*" OR "spinal cord transsection*" OR "spinal cord transverse lesion*" OR "Spinal Cord Trauma*" OR "tic doloureux" OR "tic douloureux" OR "transverse cord lesion*" OR "transverse lesion*" OR "transverse spinal cord lesion*" OR "Traumatic Myelopath*" OR "type ii complex regional pain syndrome*")

3 TITLE-ABS-KEY((evidence W/1 based) or (meta W/1 analys*) or (systematic* W/3 review*) or guideline* or (control* W/3 study) or (control* W/3 trial) or (randomized W/3 study) or (randomized W/3 trial) or (randomised W/3 study) or (randomised W/3 trial) or "pragmatic clinical trial" or (doubl* W/1 blind*) or (doubl* W/1 mask*) or (singl* W/1 blind*) or (singl* W/1 mask*) or (tripl* W/1 blind*) or (tripl* W/1 mask*) or (trebl* W/1 blind*) or (trebl* W/1 mask*) or "latin square" or placebo* or nocebo* or multivariate or "comparative study" or "comparative survey" or "comparative analysis" or (intervention* W/2 study) or (intervention* W/2 trial) or "cross-sectional study" or "cross-sectional analysis" or "cross-sectional survey" or "cross-sectional design" or "prevalence study" or "prevalence analysis" or "prevalence survey" or "disease frequency study" or "disease frequency analysis" or "disease frequency survey" or crossover or "cross-over" or cohort* or "longitudinal study" or "longitudinal survey" or "longitudinal analysis" or "longitudinal evaluation" or longitudinal* or ((retrospective or "ex post facto") W/3 (study or survey or analysis or design)) or retrospectiv* or "prospective study" or "prospective survey" or "prospective analysis" or prospectiv* or "concurrent study" or "concurrent survey" or "concurrent analysis" or "clinical study" or "clinical trial" or "case control study" or "case base study" or "case referrent study" or "case referent study" or "case referent study" or "case compeer study" or "case comparison study" or "matched case control" or "multicenter study" or "multi-center study" or "odds ratio" or "confidence interval" or "change analysis" or ((study or trial or random* or control*) and compar*))

4 1 and 2 and 3

5 TITLE-ABS-KEY((alpaca OR alpacas OR amphibian OR amphibians OR animal OR animals OR antelope OR armadillo OR armadillos OR avian OR baboon OR baboons OR beagle OR beagles OR bee OR bees OR bird OR birds OR bison OR bovine OR buffalo OR buffaloes OR buffalos OR "c elegans" OR "Caenorhabditis elegans" OR camel OR camels OR canine OR canines OR carp OR cats OR cattle OR chick OR chicken OR chickens OR chicks OR chimp OR chimpanze OR chimpanzees OR chimps OR cow OR cows OR "D melanogaster" OR "dairy calf" OR "dairy calves" OR deer OR dog OR dogs OR donkey OR donkeys OR drosophila OR "Drosophila melanogaster" OR duck OR duckling OR ducklings OR ducks OR equid OR equids OR equine OR equines OR feline OR felines OR ferret OR ferrets OR finch OR finches OR fish OR flatworm OR flatworms OR fox OR foxes OR frog OR frogs OR "fruit flies" OR "fruit fly" OR "G mellonella" OR "Galleria mellonella" OR geese OR gerbil OR gerbils OR goat OR goats OR goose OR gorilla OR gorillas OR hamster OR hamsters OR hare OR hares OR heifer OR heifers OR horse OR horses OR insect OR insects OR jellyfish OR kangaroo OR kangaroos OR kitten OR kittens OR lagomorph OR lagomorphs OR lamb OR lambs OR llama OR llamas OR macaque OR macaques OR macaw OR macaws OR marmoset OR marmosets OR mice OR minipig OR minipigs OR mink OR minks OR monkey OR monkeys OR mouse OR mule OR mules OR nematode OR nematodes OR octopus OR octopuses OR orangutan OR "orang-utan" OR orangutans OR "orang-utans" OR oxen OR parrot OR parrots OR pig OR pigeon OR pigeons OR piglet OR piglets OR pigs OR porcine OR primate OR primates OR quail OR rabbit OR rabbits OR rat OR rats OR reptile OR reptiles OR rodent OR rodents OR ruminant OR ruminants OR salmon OR sheep OR shrimp OR slug OR slugs OR swine OR tamarin OR tamarins OR toad OR toads OR trout OR urchin OR urchins OR vole OR voles OR waxworm OR waxworms OR worm OR worms OR xenopus OR "zebra fish" OR zebrafish) AND NOT (human OR humans or patient or patients))

6 4 and not 5

7 TITLE-ABS-KEY(case W/3 report)

8 6 and not 7

9 DOCTYPE(ed) OR DOCTYPE(bk) OR DOCTYPE(er) OR DOCTYPE(no) OR DOCTYPE(sh)

10 8 and not 9

11 INDEX(embase) OR INDEX(medline) OR PMID(0* OR 1* OR 2* OR 3* OR 4* OR 5* OR 6* OR 7* OR 8* OR 9*)

12 10 and not 11
